# Integrated metabolomic and transcriptomic analysis reveals the role of phenylpropanoid biosynthesis pathway in tomato roots during salt stress

**DOI:** 10.3389/fpls.2022.1023696

**Published:** 2022-12-08

**Authors:** Chunping Jia, Bin Guo, Baike Wang, Xin Li, Tao Yang, Ning Li, Juan Wang, Qinghui Yu

**Affiliations:** ^1^ College of Life Science and Technology, Xinjiang University, Urumqi, China; ^2^ Institute of Horticulture Crops, Xinjiang Academy of Agricultural Sciences, Key Laboratory of Genome Research and Genetic Improvement of Xinjiang Characteristic Fruits and Vegetables, Urumqi, China; ^3^ College of Computer and Information Engineering, Xinjiang Agricultural University, Urumqi, China

**Keywords:** tomato roots, integrated multi-omics analysis, phenylpropanoid biosynthesis, regulatory network, transcription factors, salt stress

## Abstract

As global soil salinization continues to intensify, there is a need to enhance salt tolerance in crops. Understanding the molecular mechanisms of tomato (*Solanum lycopersicum*) roots’ adaptation to salt stress is of great significance to enhance its salt tolerance and promote its planting in saline soils. A combined analysis of the metabolome and transcriptome of *S. lycopersicum* roots under different periods of salt stress according to changes in phenotypic and root physiological indices revealed that different accumulated metabolites and differentially expressed genes (DEGs) associated with phenylpropanoid biosynthesis were significantly altered. The levels of phenylpropanoids increased and showed a dynamic trend with the duration of salt stress. Ferulic acid (FA) and spermidine (Spd) levels were substantially up-regulated at the initial and mid-late stages of salt stress, respectively, and were significantly correlated with the expression of the corresponding synthetic genes. The results of canonical correlation analysis screening of highly correlated DEGs and construction of regulatory relationship networks with transcription factors (TFs) for FA and Spd, respectively, showed that the obtained target genes were regulated by most of the TFs, and TFs such as MYB, Dof, BPC, GRAS, and AP2/ERF might contribute to the regulation of FA and Spd content levels. Ultimately, FA and Spd attenuated the harm caused by salt stress in *S. lycopersicum*, and they may be key regulators of its salt tolerance. These findings uncover the dynamics and possible molecular mechanisms of phenylpropanoids during different salt stress periods, providing a basis for future studies and crop improvement.

## Introduction

Soil salinization is a land degradation process that restricts plant growth and grain yield and has become a global problem that continues to deteriorate (Yuan et al., 2016; Landi et al., 2017; Gu et al., 2022). Currently, besides the original salinized soils, which account for at least 7% of the world’s entire land area, irrigated lands and agricultural lands are increasingly subject to salinization, and their affected area is expected to double by 2050 (Rengasamy, 2006; Chele et al., 2021). At this point, grain production is required to increase by 70-110% to largely satisfy the food requirement of the rapidly growing global population (Tilman et al., 2011; Butcher et al., 2016; Molotoks et al., 2020). Salt stress has become one of the main constraints on agricultural production (Zhu, 2002; Munns and Tester, 2008; Behera et al., 2021). With the premise that it is difficult to continue to expand the arable land area, it is particularly crucial to explore salt resistance mechanisms, excavate the salt tolerance genes, and eventually enhance the salt resistance of crops (Yamaguchi and Blumwald, 2005; van Zelm et al., 2020).

Tomato (*Solanum lycopersicum*) is an essential nutritious vegetable crop that is widely cultivated around the world. However, its growth and productivity are vulnerable to salt stress due to long-term artificially directed selection (Zhu et al., 2014; Zhang et al., 2017). For example, high salt environments can produce numerous adverse impacts such as decreased germination rate, inhibited growth and development, and reduced yield and quality (Cuartero et al., 2006; Tanveer et al., 2019).

During growth and development, in reaction to osmotic stress, oxidative stress, ion toxicity, and nutrient imbalance caused by harsh hypersaline environments, plants often adapt to such changes by adopting a variety of strategies including signal transduction, regulation of specific gene expression, ion transport and uptake, protein synthesis and turnover, and carbohydrate and energy metabolism (Hirayama and Shinozaki, 2010; Zhu, 2016; Ismail and Horie, 2017; Afzal et al., 2022). By generating a multitude of physiological responses in plants, such as establishing antioxidant defense and inducing enzyme scavenging systems, including antioxidant enzymes such as peroxidase (POD), superoxide dismutase (SOD), catalase (CAT), and ascorbate peroxidase (APX) to inhibit the outbreak of reactive oxygen species (ROS) and reduce the damage caused to fundamental substances such as DNA, proteins, and lipids damage. At the same time, K^+^ (an indispensable component for plant growth) and Na^+^ content in the cytoplasm are dynamically regulated to maintain a normal K^+^/Na^+^ ratio level, and mitigate the adverse effects of cell damage and decreased productivity caused by inhibited K^+^ uptake (Miller et al., 2010; Brini and Masmoudi, 2012; Yang and Guo, 2018). Meanwhile, high salt environments are also accompanied by changes in many metabolic processes and metabolites, such as the accumulation of proline (Pro), betaine, flavonoids, and sucrose engaged in the osmoregulation of plants during salt stress, which may enhance the tolerance of plants against salt stress (Behr et al., 2017; Esfandiari Ghalati et al., 2020; Zhang et al., 2021). Changes in the content of fatty acids, organic acids, amino acids, and phytohormones can ameliorate plant growth to adapt to salt stress (Kumari and Parida, 2018; Batista-Silva et al., 2019).

Currently, genomic technologies have become increasingly systematic and accessible. For example, transcriptomics and metabolomics analysis approaches have been effectively used to investigate the reaction mechanisms of gene expression and metabolite accumulation under salt stress, and an increasing number of molecular mechanisms have been uncovered (Ma et al., 2020; Xu et al., 2021; Qin et al., 2022). Besides, roots are the main carriers of nutrient and water uptake by plants and are the first organs to be damaged by salinity and the most sensitive against salt stress. This means that the effective response capacity of plants under salt stress depends on the roots (Yang and Guo, 2018; Deolu-Ajayi et al., 2019; Nakamura et al., 2020). In recent years, the results of studies that integrated physiological, transcriptional, and metabolic analyses to reveal the molecular mechanisms of salt tolerance in soybean (*Glycine max*) roots have demonstrated that salt tolerant varieties have higher rates of nitrogen (N) uptake and assimilation, an increased amino acid accumulation and more rapid tricarboxylic acid cycle activity in response to salt stress, which contributes to their improved adaptation under salt stress (Jin et al., 2021). The findings of combined transcriptional and metabolic analyses to reveal the effects of the phenylpropanoid biosynthesis pathway during salt resistance of fabaceae (*Sophora alopecuroides*) suggest that lignans and flavonoids are probably engaged in the scavenging of ROS from *S. alopecuroides* roots, mitigating the damage induced by salt stress (Zhu et al., 2021). The results of transcriptional and metabolomic analyses to uncover mechanisms of salt acclimation in sugar beet (*Beta vulgaris*) roots indicate that carbon (C) and N metabolism is changed in response to salt stress. The N metabolism takes a primary effect in the late period under salt stress. Allantoin, which is engaged in the purine metabolic pathway is probably a critical regulator of salt resistance in *B. vulgaris* (Liu et al., 2020). Likewise, the results based on transcriptional and metabolomic analysis of key biological pathways indicate that an essential metabolic pathway in canola (*Brassica napus*) roots in response to salt stress is lipid metabolism. The roots of *B. napus* seedlings can respond to salt stress *via* lipid metabolism genes and metabolites (Wang et al., 2022b).

Presently, studies on the combined analysis of the transcriptome and metabolome of *S. lycopersicum* in reaction to salt stress have been described in leaves (Mellidou et al., 2021), but studies on the molecular mechanisms of salt tolerance in *S. lycopersicum* roots using a multi-omics approach have rarely been reported, and the understanding of the physiological and molecular mechanisms occurring in *S. lycopersicum* in response to salt stress is still incomplete. Here, we performed integrated physiological, transcriptomic, and metabolomic analyses of *S. lycopersicum* roots to explore the mechanisms of relevant gene expression and metabolite accumulation during different salt stress periods, to determine critical regulatory genes, metabolites, and metabolic pathways that may be related to salt tolerance, and to further identify potential candidate genes and transcription factors (TFs) by correlation analysis of transcripts and metabolites. This work gives valuable insights for enhancing salt resistance in *S. lycopersicum*, helps to deepen the comprehension of the molecular regulatory mechanisms of the salt stress response, and lays the foundation for future cultivation breeding and genetic improvement.

## Materials and methods

### Plant growth, treatment, and harvested samples

Tomato (*S. lycopersicum* cv. Alisa Craig) seeds were planted in a 3:1 (w/w) mixture of nutrient soil and vermiculite and grown in an artificial greenhouse at a controlled temperature of 24°C ± 2°C, relative humidity of 60% ± 10%, and a photoperiod of 16 h/8 h (daytime: 08:00 to 00:00). After 4 weeks, the seedling roots were rinsed in running water and moved to pots filled with 1/4 Hoagland’s nutrient solution (NSP1020-50L, Coolaber, Beijing, China) for hydroponics. After 1 week of recovery, the seedlings were moved to pots filled with 1/2 Hoagland’s nutrient solution (NSP1020-50L, Coolaber, Beijing, China) with 200 mM NaCl for salt stress treatment. Samples were collected at 0 h, 1 h, 12 h, and 24 h post-treatment (roots of 10 uniformly growing plants were collected from each sample, mixed, and divided into two parts for transcriptomic and widely targeted metabolomic analyses), and three biological replicates were set up for each treatment. All samples were rapidly frozen in liquid N and stored in an ultra-low temperature refrigerator at -80°C.

### Detection of physiological indices in *S. lycopersicum* roots

According to the manufacturer’s instructions, nine test kits (H2O2-1-Y, SA-1-G, POD-1-Y, SOD-1-Y, CAT-1-Y, APX-1-W, GR-1-W, PRO-1-Y, and MDA-1-Y, Comin, Suzhou, China) were used sequentially to detect hydrogen peroxide (H_2_O_2_), superoxide anion (O_2_^-^), POD, SOD, CAT, APX, glutathione reductase (GR), Pro, and malondialdehyde (MDA) content/activity, and three replicates were performed.

### Widely targeted metabolic profiling

To investigate the metabolite changes in *S. lycopersicum* roots under different treatment periods of salt stress, we performed metabolic analysis on samples from three biological replicates under each treatment period. Sample preparation, metabolite extraction, and analysis were provided by Metware Biotechnology Co. Ltd (Wuhan, China), modified according to the manufacturer’s protocol. The pulverized 100 mg of lyophilized sample powder was transferred to a pre-cooled centrifuge tube, 1.2 mL of 70% methanol solution was added, vortex shaken 6 times, and the extraction was performed overnight at 4°C. The above extracts were centrifuged at 12,000 rpm for 10 min and the supernatant was transferred to a microporous membrane (0.22 µm pore size) for filtration and then stored in the autosampler vials. Then, they were analyzed using an ultra performance liquid chromatography (UPLC) - tandem mass spectrometry (MS/MS) data acquisition system (UPLC, SHIMADZU Nexera X2, https://www.shimadzu.com.cn/; MS, Applied Biosystems 4500 Q TRAP, http://www.appliedbiosystems.com.cn/). The system is controlled using Analyst 1.6.3 software (AB Sciex) and is equipped with an electron spray ionization (ESI) turbo ion spray interface that operates in positive and negative ion mode, alternating an ESI-triple quadrupole-linear ion trap (Q TRAP) - MS scan of the effluent. Aqueous solutions of 10 and 100 µmol/L of polypropylene glycol were used for instrument tuning and mass calibration, respectively. Liquid phase conditions: equal volumes (4 µL) were injected into the column (Agilent SB-C18, 1.8 µm, 2.1 mm × 100 mm, Shanghai, China). Mobile phase A (0.1% formic acid in ultrapure water) and mobile phase B (0.1% formic acid in acetonitrile) were used at a column temperature of 40°C and a flow rate of 0.35 mL/min. Elution gradient: the proportion of B phase was 5% for 0 min, increased linearly to 95% for 9 min and maintained at 95% for 1 min, decreased to 5% for 10-11 min and equilibrated with 5% to 14 min. ESI source operating parameters: turbo spray, spray voltage set to 5500 V (positive ion mode)/-4500 V (negative ion mode), source temperature set to 550°C, curtain gas, gas I, and gas II set to 25, 50, and 60 psi, respectively, and collision-induced ionization parameters set to high. Substance characterization was performed based on secondary spectral information through Metware Biotechnology Co. Ltd (Wuhan, China) self-built database metaware database (MWDB), and the analysis was performed using standard metabolic procedures for data preprocessing and metabolite identification, removing isotopic signals, containing K^+^ ions, Na^+^ ions, and NH4^+^ ions, and repeating signals of fragment ions that are themselves other larger molecular weight substances. The multiple reaction monitoring method was used for metabolite quantification by integrating the peak areas of the mass spectrometry peaks for all substances and correcting the integration of the mass spectrometry peaks for the same metabolite in different samples among them. After sample quality control analysis and repeat correlation assessment were completed, the samples were analyzed by univariate statistical analysis, such as *P* value, fold change, and multivariate statistical analysis, consisting of principal component analysis (PCA) and orthogonal partial least squares-discriminant analysis as well as model validation methods to obtain the dynamic profile of metabolite accumulation variances (fold change sizes were ranked from smallest to largest) and screening of different accumulated metabolites (DAMs). (fold change ≥ 2 or fold change ≤ 0.5, (variable importance in projection, VIP) ≥ 1). Determined metabolites were annotated against the kyoto encyclopedia of genes and genomes (KEGG) compound database (http://www.kegg.jp/kegg/compound/), and annotated metabolites were subsequently mapped to the KEGG pathway database (http://www.kegg.jp/kegg/pathway.html).

### RNA extraction, RNA- seq, and sequence data processing

Total RNA from root samples was extracted with RNAprep Pure Plant kit (DP441, Tiangen, China) and measured and checked for concentration and integrity using a Qubit 2.0 Fluorometer (Life Technologies, Carlsbad, CA, USA) and an Agilent Bioanalyzer 2100 system (Agilent Technologies, Palo Alto, CA, USA), respectively. The cDNA libraries were obtained by mRNA acquisition and final PCR enrichment, and the library quality control was performed to detect the insert size and accurately quantify the effective concentration (>2 nM), and after passing the library inspection, pooling was performed based on the target downstream data volume, and sequencing was performed on the Illumina platform, all of which was handed over to Metware Biotechnology Co. Ltd. (Wuhan, China) for completion. The data were subjected to strict quality control using fastp v0.19.3, and then clean reads used for follow-up analysis were acquired by checking the sequencing error rate and GC content distribution, and finally, indexes were constructed using HISAT2 v2.1.0 and compared to the reference genome (Kim et al., 2015; Chen et al., 2018). Gene matching was calculated using featureCounts v1.6.2, and the fragments per kilobase of transcript per million fragments mapped (FPKM) method was employed as a measure of gene expression profiling (Liao et al., 2014). Differential expression analysis between the two groups was performed with DESeq2 v1.22.1, with a corrected *P* value < 0.05 and |log_2_Fold Change| ≥ 1 as the threshold for significant differential expression and defined as differentially expressed genes (DEGs) (Love et al., 2014; Varet et al., 2016). Enrichment analysis was conducted according to hypergeometric tests, for the KEGG, the hypergeometric distribution test was carried out in pathway units, and for gene ontology (GO), it was based on the GO term.

### Quantitative real time polymerase chain reaction analysis

First-strand cDNA synthesis was completed with the 5×All-In-One RT MasterMix (with AccuRT Genomic DNA Removal Kit) (G492, ABM, Vancouver, Canada) following the manufacturer’s instructions. Specific primers were engineered using the national center for biotechnology information (NCBI) primer design tool (https://www.ncbi.nlm.nih.gov/tools/primer-blast/index.cgi?LINK_LOC=BlastHome), and the *Actin* (*Solyc03g078400.2*) as an internal reference control. qRT-PCR was implemented using ChamQ Universal SYBR qPCR Master Mix (Q711, Vazyme, Nanjing, China) on a LightCycler^®^ real-time fluorescent quantitative PCR system (Roche, Basel, Switzerland) using the primers presented in **Supplementary Materials Table 1**. For each sample, three technical replicates were performed to calculate the mean cycle threshold (C_t_) value. Relative expressions were calculated using the 2^-ΔΔCt^ method (Livak and Schmittgen, 2001).

### Combined analysis of transcriptomic and metabolomic

To better understand the interactions between transcriptome and metabolome in *S. lycopersicum* roots under different treatment periods of salt stress, DEGs and DAMs in different comparison groups were mapped simultaneously to the KEGG pathway database and subjected to KEGG markup language (KGML) analysis, expression correlation analysis and canonical correlation analysis (CCA) to collect information about their common pathways.

### Statistical analysis

Three independent biological replicates of each experiment were mean and analyzed. A one-way analysis of variance was conducted with GraphPad Prism for Windows (version 9.0.0, GraphPad Software, San Diego, CA, USA), and subsequently Dunnett’s multiple comparison test was applied.

## Results

### Changes in phenotypic and root physiological indices of *S. lycopersicum* under different salt stress periods

**Figure 1:**
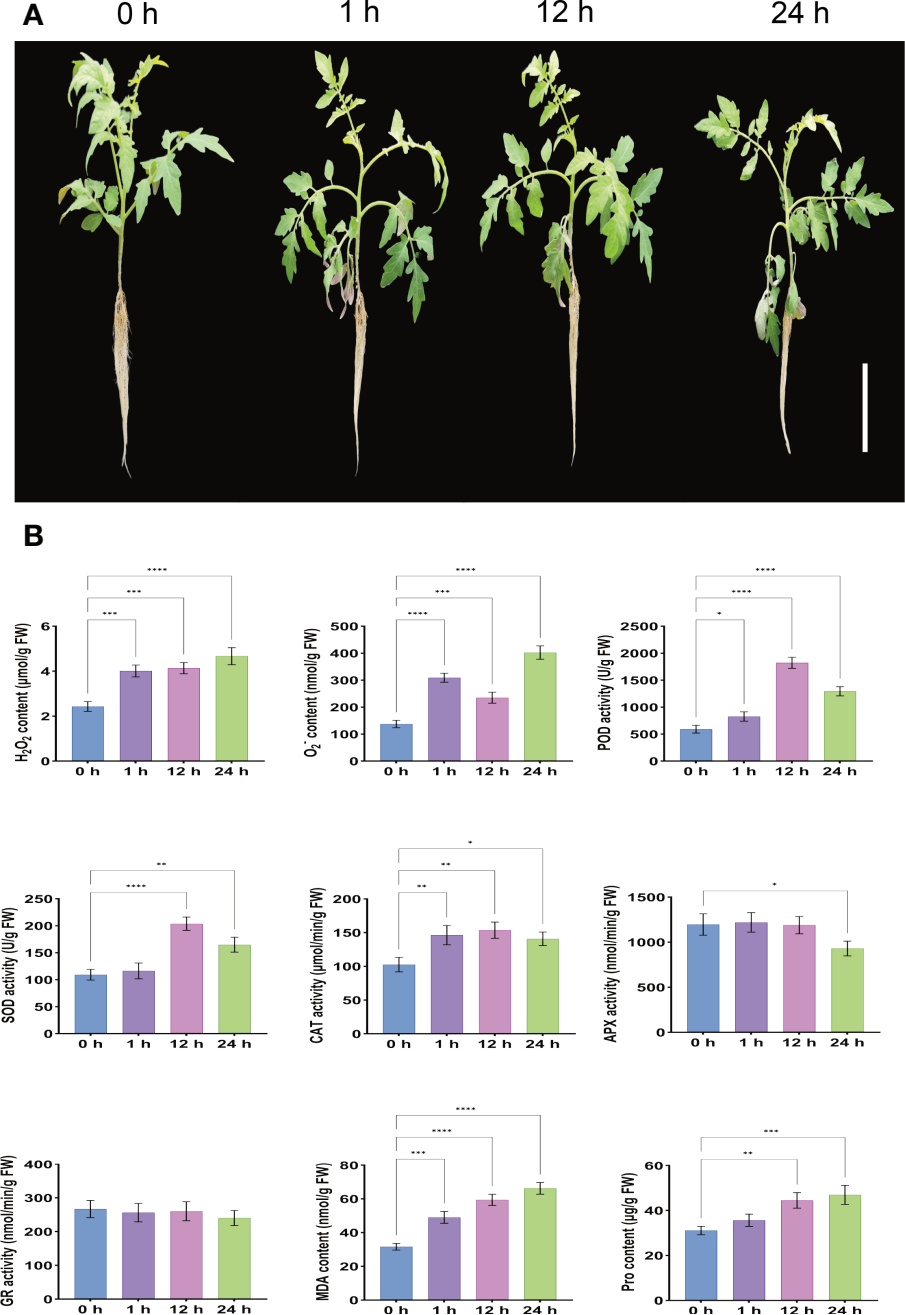
Determination of phenotypic changes and root physiological indices in *S. lycopersicum* after salt stress. **(A)** Phenotypic pictures of *S. lycopersicum* plants under different salt stress periods. **(B)** Measurement of physiological indices of *S. lycopersicum* roots during various salt stress periods. Three independent biological replicates were used to calculate the mean. Error bars indicate the standard deviation (SD) of the three biological replicates. Values indicate mean ± SD. Dunnett’s multiple comparison test was applied to verify the significance of differences (^*^ *P* < 0.05, ^**^ *P* < 0.01, ^***^ *P* < 0.001, and ^****^ *P* < 0.0001).

The morphological characteristics of the leaves can directly represent the degree of adaptation of the plant to the adversity of the state of affairs. Phenotypic analysis under salt stress showed that compared with the control (0 h), *S. lycopersicum* plants showed rapid water loss and wilting at 1 h after treatment due to reduced root osmotic water potential and absorption capacity, which inhibited normal aboveground growth and development, resulting in leaf curling and petiole softening. At 12 h after treatment, the leaf and petiole phenotypes were partially restored to normal due to the enhanced protective enzyme activity in the enzymatic defense system, and ion homeostasis. However, 24 h after treatment, salt ions were loaded into the xylem in large quantities in the roots through ion channels, carrier transport, and leakage of plastid exosomes from the roots, and were transported upward with transpiration flow to the aboveground for excessive accumulation, inhibition of enzyme activity expression and restriction of nutrient supply, resulting in more severe ionotoxic effects and increased leaf curling and petiole softening (**Figure 1A**).

In addition, roots are the main carrier of nutrients and water for plants, the main organ for nutrient assimilation and synthesis of their constituents, and the first organ to be damaged by salinity and the most susceptible to salt stress. Our results on the physiological indices of roots under different treatment periods (0 h, 1 h, 12 h, and 24 h) of salt stress showed that the contents of H_2_O_2_, O_2_^-^, and MDA increased gradually with the duration of salt stress treatment time, showing a positive correlation with highly significant differences. This indicates that the balance of the reactive oxygen metabolic system was disrupted, and the massive production of free radicals and the intensification of membrane lipid peroxidation accelerated the extent of damage to the intracellular membrane structure. The content of Pro also gradually increased with the duration of salt stress treatment time, reflecting the resistance to some extent, but there was no significant difference at 1 h after stress. The POD, SOD, and CAT are the three main antioxidant enzymes in the plant antioxidant defense system, and their activities increased significantly with the extension of salt stress treatment period and showed an overall trend of increasing and then decreasing, reaching a peak at 12 h after stress, and their activity levels can reflect the degree of plant exposure to external adversity. The APX is a type of terminal oxidase and an important antioxidant enzyme for scavenging ROS in plants. Its activity was not significantly different at 1 h and 12 after stress, but significantly decreased at 24 h. It is possible that the high concentration of H_2_O_2_ disrupted the intermediate products of APX catalytic process, which led to the inhibition of APX activity and presumably the dynamic equilibrium state between H_2_O_2_ concentration and APX expression was broken. The GR is involved in plant ascorbate- glutathione (AsA-GSH) cycle, a key antioxidant enzyme class for ROS scavenging in plants, and its activity was slightly but not significantly reduced after stress, which may have led to the reduction of GSH content and turnover rate. Considering that most of the physiological functions of the above antioxidant enzyme classes are related to a series of physiological and biochemical changes such as damage and disruption of cell membranes, disruption of cell spatial structure, and transduction of damage signals due to the effects of one (e.g. salt stress) or multiple stresses, suggesting that 24 h after stress, the oxidative defense system is affected and the ability to scavenge free radicals and H_2_O_2_ is reduced and cannot continue to act synergistically to maintain free radical content in the plant to maintain steady-state levels, already producing free radical-induced changes in plant physiological and biochemical indices (**Figure 1B**). These trends in physiological and biochemical indicators are generally consistent with the observed phenotypic changes.

### Metabolomic study of *S. lycopersicum* roots response to salt stress

#### Qualitative and quantitative analysis of metabolites

**Figure 2:**
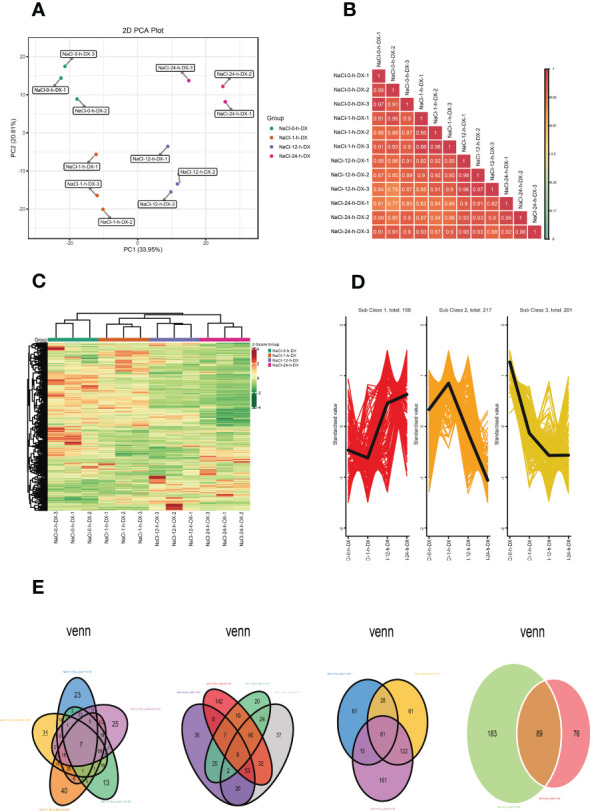
Overview of the time course (0 h, 1 h, 12 h, and 24 h) of the *S. lycopersicum* root metabolome in response to salt stress. **(A)** The plot of the overall sample PCA analysis. PC1 indicates the first principal component, PC2 indicates the second principal component, and the percentage denotes the explanation rate of this principal component for the data set, each dot in the plot indicates one sample, and samples of the identical cluster are denoted using the identical color. **(B)** Correlation plot of inter-sample. The vertical and diagonal lines represent the sample names of various samples, and various colors indicate various |r| magnitudes, the redder the color means stronger positive correlation, the greener the color means poorer correlation, and the bluer the color means stronger negative correlation, while the correlation coefficient magnitudes between two samples are marked in the squares. **(C)** Graph of the results of cluster analysis. Horizontal is the sample description, vertical is the metabolite profile, the group is the grouping, class is the substance classification, and various colors are the values derived after normalization of relative content (red means high content, green means low content). **(D)** The plot of K-means cluster analysis. The horizontal coordinate indicates the sample title, the vertical coordinate indicates the normalized metabolite relative content, the ‘Sub Class’ denotes the amount of metabolite classes with a similar trend, and ‘total’ denotes the total amount of metabolites in that class. **(E)** Venn diagram of DAMs. Each circle in the graph denotes a comparison group, and the DAMs were analyzed for five, four, three, and two comparison groups in left-to-right order, respectively. The numbers in the overlapping part of circles and circles represent the number of DAMs shared between comparison groups, and the numbers without the overlapping part represent the amount of DAMs specific to the comparison groups.

Metabolites are the basis of the phenotype of an organism and can contribute to understanding biological processes (BPs) and their mechanisms more visually and effectively. To investigate the changes of metabolites in *S. lycopersicum* roots under different periods of salt stress, we performed a widely targeted metabolic analysis. A total of 942 metabolites were detected according to the UPLC-MS/MS detection platform and a self-constructed database. First, we performed PCA on the metabolome data to get a preliminary knowledge of the overall metabolic variation among the groups and the magnitude of variation among the samples within the groups (**Figure 2A**). PC1 and PC2 accounted for 33.95% and 20.81% of the variation in metabolites of all samples, respectively, and could be well separated between groups and largely clustered together within groups, indicating overall good biological reproducibility settings within groups and large differences in metabolites between groups. Meanwhile, the replicate correlation assessment indicated that the Pearson correlation coefficient (|r|) of the within-group samples relative to the between-group samples was high (|r| > 0.8), and the closer |r| was to one, the higher the correlation between the two replicate samples, the more dependable the obtained differential metabolites were, which can be used for further analysis (**Figure 2B**). To easily visualize the variation pattern of metabolite relative contents, we adopted unit variance scaling by row to the raw relative contents of the DAMs identified by applying screening criteria and drew a hierarchical clustering heatmap (**Figure 2C**). The results showed that *S. lycopersicum* roots exhibited significant metabolic changes under salt stress, with different spatial and temporal distribution and accumulation of metabolite contents of lipids, phenolic acids, alkaloids, and flavonoids in each group of samples, which varied widely.

#### Screening and identification of DAMs

The results of DAMs screening and identification showed that 95, 163, 249, 130, 274, and 140 DAMs were detected in the six comparison groups (0 h vs. 1 h, 0 h vs. 12 h, 0 h vs. 24 h, 1 h vs. 12 h, 1 h vs. 24 h, and 12 h vs. 24 h), respectively. The largest upregulation was observed in 1 h vs. 24 h, while the smallest upregulation was observed in 12 h vs. 24 h. Notably, the largest amount of DAMs was downregulated in 1 h vs. 24 h, while the smallest number of DAMs were downregulated in 0 h vs. 1 h. We observed that after log_2_ treatment of the different multiples in each comparison group, the DAMs with the highest upregulation multiples were lysoPE 20:2 belonging to lipids in 0 h vs. 1 h and 0 h vs. 12 h, isoguanine belonging to nucleotides, and derivatives in 12 h vs. 24 h, and spermidine (Spd) belonging to alkaloids in the remaining three groups. The DAMs with the highest downregulation fold were furanodienone, which is a terpenoid in 0 h vs. 1 h, isoguanine, which is nucleotides and derivatives in 0 h vs. 12 h and 1 h vs. 12 h, and gallic acid, which is a phenolic acid in the remaining three groups (**Supplementary Figure 1**). Likewise, since DAMs had synergistic or mutually exclusive relationships, we also plotted chord diagrams of DAMs (|r| ≥ 0.8 and *P* < 0.05) for each comparison group by Pearson correlation analysis to further understand their mutual regulation during salt stress. In general, the correlations between DAMs in different comparison groups were different, and the number of positive correlations (red lines) was higher than the number of negative correlations (blue lines), i.e. synergistic is greater than mutually exclusive (**Supplementary Figure 2**).

In addition, we also investigated the K means of DAMs to survey the trend changes in the relative content of DAMs in various comparison groups (**Figure 2D**). The results showed that DAMs in sub-class three showed an all-time decreasing trend with increasing treatment time, and DAMs in sub-class one showed a decreasing trend at 0 h and an increasing trend at 12 h and 24 h. Interestingly, the trend change of DAMs in sub-class two was the opposite compared to that in sub-class one. Finally, we also plotted Venn diagrams to illustrate the number of DAMs in the different comparison groups, analyzing their intersection and peculiarities (**Figure 2E**). Among them, the total number of DAMs in the five, four, three, and two comparison groups was 7, 8, 61, and 89, respectively. It is noteworthy that the number of DAMs specific to each comparison group varied considerably, especially in the four comparison groups, the number of DAMs specific to 0 h vs. 24 h was more than 7 times higher than that of 1 h vs. 12 h.

#### KEGG pathway analysis of DAMs

**Figure 3:**
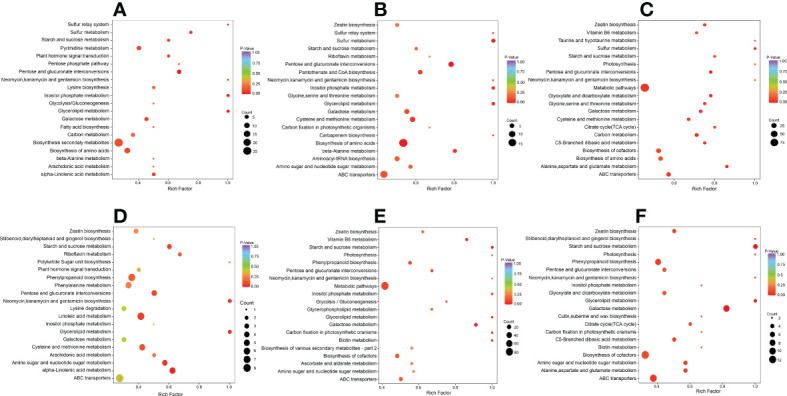
Scatter plot of DAMs for KEGG enrichment analysis. **(A)** The comparison group of 0 h vs. 1 h. **(B)** The comparison group of 0 h vs. 12 h. **(C)** The comparison group of 0 h vs. 24 h. **(D)** The comparison group of 1 h vs. 12 h. **(E)** The comparison group of 1 h *vs*. 24 h. **(F)** The comparison group of 12 h vs. 24 h. The vertical coordinate indicates the KEGG pathway. The horizontal coordinate represents the rich factor (the ratio of the amount of DAMs abundant in the pathway to the amount of annotated metabolites), the larger the rich factor, the greater the enrichment, the smaller the *P* value, and the more significant the enrichment. The larger the dot, the greater the amount of DAMs enriched in the pathway. The redder the color of the dot, the more noticeable the enrichment. We selected the 20 most significantly enriched pathway entries for display in this figure.

We carried out a KEGG enrichment analysis for DAMs in each of the six comparison groups to determine the major metabolic pathways (**Figure 3**). The outcomes showed that during the early, middle, and late stages of salt stress encountered by *S. lycopersicum* roots, i.e. 0 h vs. 1 h, 0 h vs. 12 h, and 0 h vs. 24 h comparison groups, “biosynthesis of secondary metabolites”, “pentose and glucuronate interconversions”, and “biosynthesis of amino acids” were the significant pathways generated by KEGG enrichment analysis, respectively. In the other three comparison groups, the important pathways for KEGG enrichment analysis were “alpha-Linolenic acid metabolism”, “starch and sucrose metabolism”, and “glycerolipid metabolism”. It is noteworthy that the main metabolic pathway common to all three comparison groups is “phenylpropanoid biosynthesis”.

### Transcriptomic study of tomato root response to salt stress

#### RNA sequencing and assembly of the transcriptome

**Figure 4:**
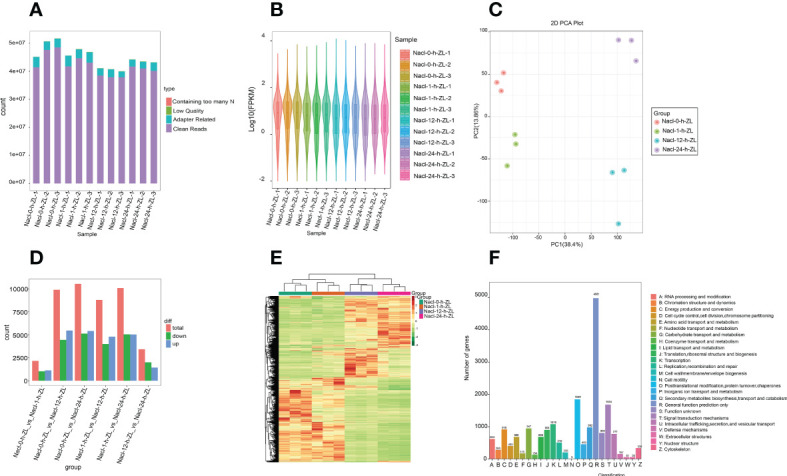
Overview of the time course (0 h, 1 h, 12 h, and 24 h) of the *S. lycopersicum* root transcriptome in response to salt stress. **(A)** Bar graph of sequencing data and quality control. Following raw data checking, sequencing error rate filtering, and GC content distribution screening, clean reads used for follow-up analysis were acquired. **(B)** Violin diagram of the amount of expression. Various colors in the graph indicate various samples, and the width of each violin graph reflects the number of genes at that expression level. **(C)** The plot of PCA. PC1 indicates the first principal component, PC2 indicates the second principal component, and the percentage represents the explanation rate of that principal component for the data set, each dot in the plot indicates one sample, and samples of the identical set are denoted using the identical color. **(D)** The statistical plot of DEGs. The red, green, and blue colors represent the total amount of DEGs, the amount of up-regulated DEGs, and the amount of down-regulated DEGs, respectively. **(E)** Clustering heatmap of DEGs. The horizontal coordinate denotes the sample titles and hierarchical clustering outcomes, and the vertical coordinate denotes the DEGs and hierarchical clustering outcomes. Red denotes high expression and green denotes low expression. **(F)** Bar graph of KOG classification. The horizontal coordinate indicates the functional classification (code) of KOG ID, and the vertical coordinate indicates the number of DEGs included, different classifications are indicated by different colors. The legend shows the code plus its functional description information.

To explore the molecular regulatory mechanism of the effect of salt stress on *S. lycopersicum* roots, root samples subjected to 200 mM NaCl were sampled at four periods (0 h, 1 h, 12 h, and 24 h). In the present study, a total of 12 samples were sequenced and 75.59 Gb of clean data were obtained by eliminating the low-quality data, and the clean data of each sample achieved 5 Gb. The distribution of Q20 base percentages ranged from 97.41% to 98.05%, Q30 base percentages ranged from 92.72% to 94.22%, and GC content ranged from 41.72% to 48.5%. It signifies the high quality of the transcriptome data (**Figure 4A**). The overall distribution of sample gene expressions showed the distribution status and probability density, and the protein-coding gene expression level FPKM values spanned multiple orders of magnitude, from 10^-2^ to 10^4^, visually comparing the overall gene expression levels of different samples (**Figure 4B**). The PCA results showed that PC1 and PC2 accounted for 38.4% and 13.86% of the variation in gene expression of all samples, respectively, which could clearly distinguish the different groups (**Figure 4C**).

#### Identification and characterization of DEGs

To provide a better demonstration of the changes in gene expression in *S. lycopersicum* roots under different treatment periods of salt stress, we used DESeq2 v1.22.1 for differential expression analysis between the two groups with the threshold set to a corrected *P* value < 0.05 and |log_2_Fold Change| >= 1. The histogram results of DEGs between the different groups (**Figure 4D** **),** showed that the largest amount of DEGs was observed in the 0 h vs. 12 h comparison group, with 5,460 up-regulated DEGs and 4,443 down-regulated DEGs. The number of DEGs in the 0 h vs. 1 h comparison group was the lowest, with 1,122 up-regulated DEGs and 1,032 down-regulated DEGs. The number of up-regulated and down-regulated DEGs in the 0 h vs. 1 h and 12 h vs. 24 h comparison groups ranged from 1,000 to 2,000, while the remaining other comparison groups ranged from 4,000 to 5,500. The above results showed that the transcripts varied greatly under different periods of salt stress, and there were significant differences in DEGs between the various comparison groups. Meanwhile, to judge the expression patterns of DEGs under different treatment periods, we took the DEGs of all comparison groups and set them together as differential gene sets for hierarchical clustering analysis, normalized the data using Z score, and drew a clustering heatmap (**Figure 4E**), the results indicated that genes with the identical or similar expression patterns under different treatment periods were clustered into classes, and these similar genes may have similar functions. Intriguingly, the expression patterns of DEGs in the 0 h vs. 1 h and 12 h vs. 24 h comparison groups showed opposite trends.

In addition, we also used diamond software to match DEGs to the clusters of orthologous groups of proteins (COG) database (https://www.ncbi.nlm.nih.gov/COG/), and then extracted the corresponding annotation information based on the database protein IDs, afterwards, extracted the annotations of the eukaryotic-specific version of the COG (KOG) database were obtained (**Figure 4F**). The findings suggest that the number of differential genes included in each KOG functional category was different, except for the differential genes annotated to general function prediction only and function unknown, the least number of differential genes annotated to cell motility was five, and conversely, the most were posttranslational modification, protein turnover, and chaperones, containing 1,848.

Finally, to investigate the trend change of DEGs, we normalized the FPKMs of all differential gene concatenations using R language and then did a K means cluster analysis (**Supplementary Figure 3**). The outcomes indicated that with the increase in treatment time, the DEGs in sub-class one and sub-class four showed a continuous decreasing trend, those in sub-class three and sub-class five showed an increasing trend, and those in sub-class two and sub-class six showed an increasing and then decreasing trend, reaching the peak at 12 h and 1 h, respectively, and this situation that the same class of genes had similar trends under different treatment periods implied that they might have similar functions.

#### GO enrichment and the KEGG pathway analysis of DEGs

**Figure 5:**
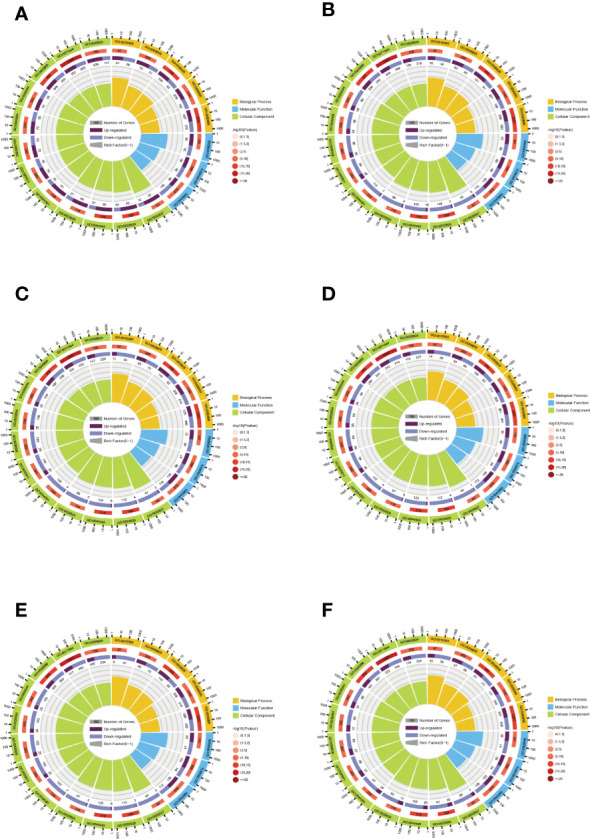
Enrichment circle plots for different comparison groups. **(A)** The comparison group of 0 h vs. 1 h. **(B)** The comparison group of 0 h vs. 12 h. **(C)** The comparison group of 0 h vs. 24 h. **(D)** The comparison group of 1 h vs. 12 h. **(E)** The comparison group of 1 h vs. 24 h. **(F)** The comparison group of 12 h vs. 24 h. There are four loops in the enrichment circle diagram from outside to inside, the first loop is the enriched category and the outside of the loop is a coordinate scale for the amount of genes, different colors indicate various categories. The second loop shows the amount and *P* value of that classification in the background genes. The longer the bar means more genes and the redder the color means smaller the value. The third loop shows the bar of the proportion of up-and down-regulated genes, with different colors representing the amount of up-and down-regulated genes, respectively. The fourth loop shows the rich factor value (the amount of foreground genes divided by the amount of background genes in that classification) for each classification, with each cell of the background auxiliary line indicating 0.1.

GO is divided into three parts: molecular functions (MFs), BPs, and cellular compositions (CCs). GO enrichment analysis can better qualify and describe gene and protein functions, and the distribution in GO is an important proof to elucidate the sample differences in gene functions. Z score bubble plots and circle plots of enrichment analysis show that the top 20 GO terms in the six comparison groups are the highest in CC (**Supplementary Figure 4**). The top GO terms in BP, CC, and MF were “response to abiotic stimulus” (GO: 0009628), “cell periphery” (GO: 0071944), and “protein serine/threonine kinase activity” (GO: 0004674). In addition, “response to stimulus” (GO: 0050896) and “galacturonan metabolic process” (GO: 0010393) had the highest and lowest number of background genes and proportion of up- and down-regulated genes, respectively (**Figure 5**).

To further explore the biological roles of DEGs, we labeled different numbers of DEGs in each comparison group in the KEGG database, thus analyzing the KEGG pathways of DEGs in different comparison groups (**Supplementary Figure 5**). The results showed that among the top 20 enrichment pathways in all comparison groups at the early, middle, and late periods of salt stress in *S. lycopersicum* roots, “phenylpropanoid biosynthesis”, “plant-pathogen interaction”, “MAPK signaling pathway-plant”, “plant hormone signal transduction”, and “biosynthesis of secondary metabolites” were the five most important pathways. These pathways offer novel insights into further exploration of salt resistance genes in *S. lycopersicum*. Notably, the “phenylpropanoid biosynthesis” pathway was almost always the most enriched in each comparison group, suggesting its significance in the reaction to salt stress in *S. lycopersicum* roots.

#### Combined transcriptome and metabolome analysis of *S. lycopersicum* root response to salt stress

**Figure 6:**
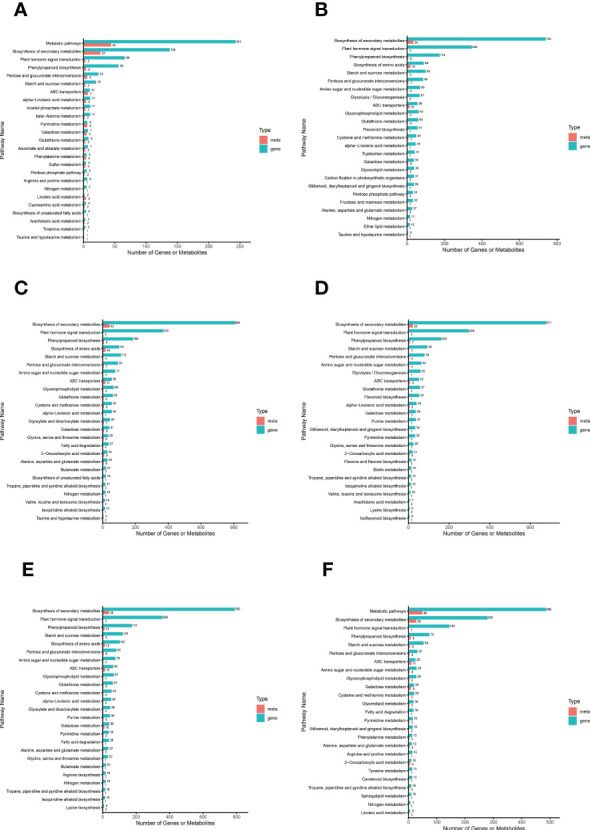
Bar graph of combined transcriptome and metabolome analysis. **(A)** The comparison group of 0 h vs. 1 h. **(B)** The comparison group of 0 h vs. 12 h. **(C)** The comparison group of 0 h vs. 24 h. **(D)** The comparison group of 1 h vs. 12 h. **(E)** The comparison group of 1 h vs. 24 h. **(F)** The comparison group of 12 h vs. 24 h. The horizontal coordinates in the bar graph represent the amount of DAMs and DEGs enriched to the pathway, the vertical coordinates represent the KEGG pathway name, and the red and green bars represent the metabolome and transcriptome, respectively.

We plotted a bar chart of the transcriptome and metabolome jointly enriched to the KEGG pathway to visualize the number of DAMs and DEGs enriched to a particular pathway (**Figure 6**). The results showed that among the top 25 pathways in terms of *P* value in all comparison groups, the top major pathways were “biosynthesis of secondary metabolites”, “plant hormone signal transduction”, and “phenylpropanoid biosynthesis”. It is evident that these three pathways have the greatest impact on *S. lycopersicum* roots during salt stress and are closely related to salt tolerance in *S. lycopersicum*. It is also implied that with the duration of salt stress, secondary metabolic pathways progressively dominate the response of *S. lycopersicum* to salt stress. To study the interactions between transcriptome and metabolome more conveniently and systematically, we obtained the network relationships of gene products and metabolites using KGML files (containing both the relationships of graphical objects in the KEGG pathway as well as the information of direct homologous genes in the KEGG GENES database) in a sub repository of the KEGG database (**Supplementary Figure 6**).

In addition, we show the differential multiplicity of substances with |r| > 0.80 and a *P* value < 0.05 in each differential comparison group by a nine-quadrant plot (black dashed line, left to right and top to bottom, sequentially grouped into quadrants one-nine) (**Supplementary Figure 7**). Compared to the remaining comparison groups, the range of genes up- and down-regulated or unchanged gene expression was greater in the 0 h vs. 1 h and 12 h vs. 24 h comparison groups with non-differential gene and metabolite expression in quadrant five and unchanged metabolite expression in quadrants two, four, six, and eight. Among all comparison groups, the pattern of differential expression of genes and metabolites in quadrants three and seven was consistent, indicating that genes and metabolites are positively correlated and that changes in metabolites are probably positively regulated by genes. The opposite patterns of differential gene and metabolite expression in quadrant one and quadrant nine suggest that genes and metabolites have a non-consistent regulatory trend and that alterations in metabolites may be negatively regulated by genes.

#### Identification of genes and metabolites related to the phenylpropanoid biosynthesis pathway

**Figure 7:**
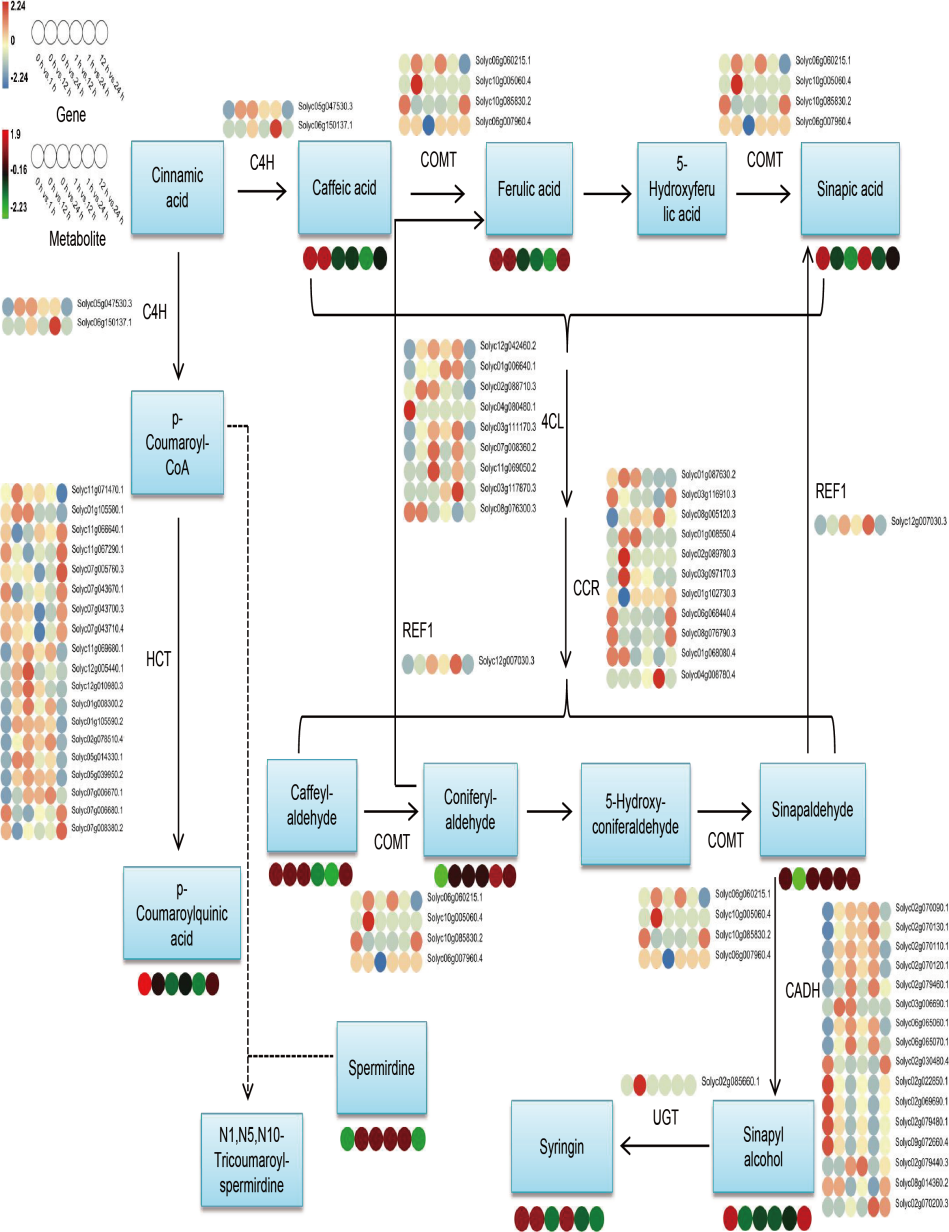
Overview map of the relationship between DEGs and DAMs within the phenylpropanoid biosynthesis pathway in *S. lycopersicum* roots under salt stress. The colored circles at the bottom show the heatmap of expression of key metabolites and transcripts associated with phenylpropanoid biosynthesis in different comparison groups. Key enzyme gene abbreviations: C4H, Cinnamate 4-monooxygenase; COMT, Caffeic acid 3-O-methyltransferase; CCR, Cinnamoyl-CoA reductase; 4CL, 4-coumarate–CoA ligase; HCT, Shikimate O-hydroxycinnamoyltransferase; REF1, Coniferyl-aldehyde dehydrogenase; UGT, Coniferyl-alcohol glucosyltransferase; CADH, Cinnamyl-alcohol dehydrogenase.

Considering the results of metabolomics, transcriptomic, and combined analyses all indicate that DEGs and DAMs are remarkably abundant in the phenylpropanoid biosynthesis pathway and that phenylpropanoids have significant contributions to plant growth and development and in responding to stresses of adversity. Therefore, we selected this pathway for further analysis. The findings of expression investigations of phenylpropanoid biosynthesis-related DEGs and DAMs in *S. lycopersicum* roots under different treatment periods of salt stress showed that (**Figure 7**), a total of 62 DEGs and 10 DAMs were characterized, and we found that upstream of the pathway, most genes encoding 4CL, CCR, and HCT had the highest number of up- and down-regulation in different comparison groups, while downstream, genes encoding CADH had the highest number of up- and down-regulation in different comparison groups. REL1 enzymatically cleaves pineal and mustard aldehyde to generate ferulic and mustardic acids, with *Solyc12g007030.3* having the highest expression in the 1 h vs. 24 h comparison group. UGT catalyzes the generation of lilacoside from mustard alcohol, with *Solyc02g085660.1* having the highest expression in the 0 h vs. 12 h comparison group. The accumulation of DAMs within the phenylpropanoid biosynthesis pathway in the different comparison groups was in agreement with the expression pattern of some DEGs, while the inconsistency may be the result of gene co-regulation.

**Figure 8:**
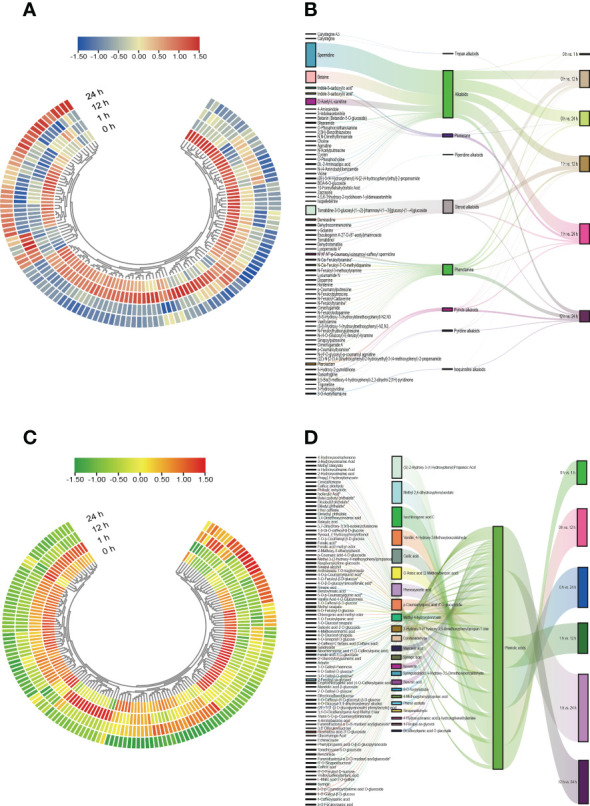
Heatmap and Sankey diagram of alkaloids and phenolic acids class of substances. **(A)** Heatmap of the changes of alkaloids in *S. lycopersicum* roots under different salt stress treatment periods. **(B)** Sankey diagram of mobility of alkaloids in *S. lycopersicum* roots in comparison groups with different salt stress treatments. **(C)** Heatmap of the changes of phenolic acids in *S. lycopersicum* roots under different salt stress treatment periods. **(D)** Sankey diagram of the mobility of phenolic acids in *S. lycopersicum* roots in comparison groups with different salt stress treatments. Heatmap is used to map each value in the data matrix to a color display according to a certain pattern, and the color change is used to visualize the data. Sankey diagrams were used to describe the flow from one set of values to another, with squares representing metabolites, classification, comparison groups, etc., and bars representing the mobility between them, with the width of the bars proportional to the size of the mobility.

We further used the quantitative values of genes and metabolites in all samples and calculated their |r| employing the cor function in R. The correlations between metabolites and genes in the phenylpropanoid biosynthesis pathway with |r| > 0.8 and *P* < 0.05 were represented by network plots (**Supplementary Figure 8** and **Supplementary Table 2**). The findings of the correlation analysis suggested that the DEGs belonging to the two major groups of alkaloids and phenolic acids, respectively, among the phenylpropane compounds were closely related to the DAMs. We plotted heatmap and Sankey diagrams to present the dynamic accumulation and the flow of one set of values to another for alkaloids and phenolic acids across treatment periods and comparison groups (**Figure 8**, **Supplementary Figure 9**, and **Supplementary Figure 10**), and the results showed that the dynamic accumulation of all alkaloids and phenolic acids varied significantly in different treatment periods and in different comparison groups. Within the Sankey diagram, different lines represent different flow diversions and the width of the line represents the size of the data represented by this branch. The baseline data values for alkaloids and phenolic acids varied considerably between the different comparison groups and were the highest in the 1 h vs. 24 h comparison group and the lowest in the 0 h vs. 1 h comparison group.

**Figure 9:**
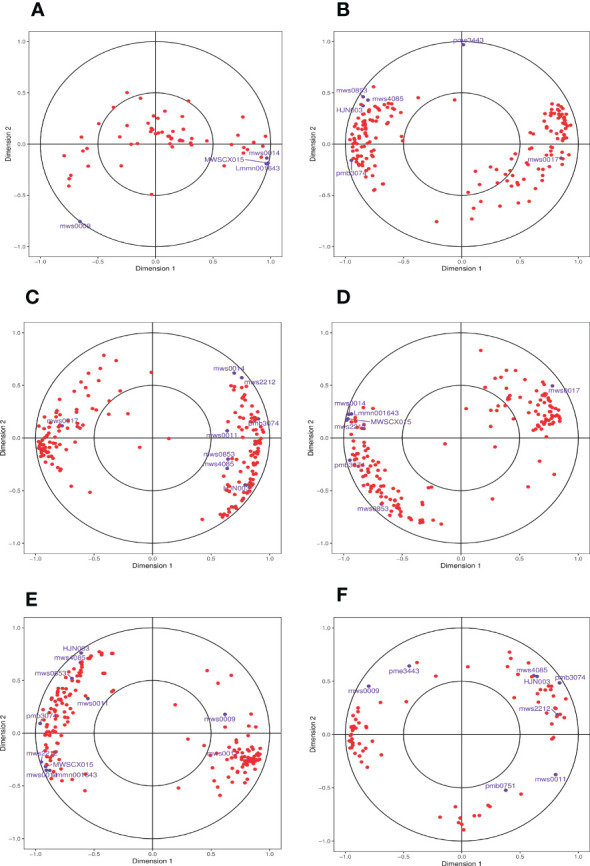
Graph of CCA of DEGs and DAMs in the phenylpropanoid biosynthesis pathway. **(A)** The comparison group of 0 h vs. 1 h. **(B)** The comparison group of 0 h vs. 12 h. **(C)** The comparison group of 0 h vs. 24 h. **(D)** The comparison group of 1 h vs. 12 h. **(E)** The comparison group of 1 h vs. 24 h. **(F)** The comparison group of 12 h vs. 24 h. Four regions are distinguished by crosses in the figure, and the longer the distance from the origin and the nearer to each other within the same region, the higher the correlation. Metabolites are labeled in purple and genes are labeled in red. If there are too many substances in a certain category, they are shown with dots instead to avoid text overlap.

Finally, we used CCA, a multivariate statistical analysis approach, to reflect the whole correlation between the two groups of indices utilizing the correlation between the combined pairs of variables (**Figure 9**). CCA of the phenylpropanoid biosynthesis pathway in the different comparison groups identified DEGs and DAMs within the identical area, the longer the distance from the origin, the nearer to each other, i.e., the stronger correlation. We found that the log_2_FC of metabolite mws0014 (ferulic acid, FA) reached 1.18 in the 0 h vs. 1 h comparison group at the beginning of salt stress compared with the control 0 h, which presented a significant up-regulation relationship and was consistent with the principle that it was far from the origin and close to the DEGs. The association was high. We adopted the principle that the two exhibited an up-regulatory relationship and high correlation for analysis and screening and found that, when compared with the control (0 h), the log_2_FC of the metabolite mws0014 (FA) reached 1.18 in the 0 h vs. 1 h comparison group at the beginning of salt stress, showing a significant up-regulation relationship and a high correlation following the principle of being far from the origin and close to each other with DEGs. Meanwhile, in both the 0 h vs. 12 h comparison group at mid-salt stress and the 0 h vs. 24 h comparison group at late salt stress, only the metabolite mws0017 (Spd) had a log_2_FC of 16.87 and 16.64, respectively, showed a significant up-regulation relationship and a high correlation following the principle of being far from the origin and close to each other with DEGs (**Supplementary Table 3**).

#### Construction of regulatory networks for gene expression and mining of TFs with potential regulatory relationships in Spd and FA

Spd is an important growth regulator in plants with important physiological activities and is widely participated in plant stress response to adversity. The majority of the FA binds to lignin, polysaccharides, and proteins in the cell through ester and ether bonds to become the backbone of the cell wall. It makes the cell wall strong and is an important molecular basis for the formation of cell wall resistance barriers to degradation. To explore the regulatory network of related gene expression in *S. lycopersicum* roots under different treatment periods of salt stress, the correlations between Spd and genes in the phenylpropanoid biosynthesis pathway with |r| > 0.8 and p < 0.05 were screened and 24 target genes were obtained. The correlations between FA and genes were screened in the same way, and 20 target genes were obtained (**Supplementary Table 4**).

Based on the regulation prediction tool (infers potential regulatory interactions between TFs and input genes, and finds the TFs which possess over-represented targets in the input gene set) in the plant transcriptional regulatory map (http://plantregmap.gao-lab.org/index.php) website, the promoter sequences 2,000 bp upstream of the start codon of total target genes were analyzed. By adjusting the parameter settings to background positions corresponding to the transcriptional start site of genes: -500 bp to +100 bp and threshold (for binding site prediction) *P* value ≤ 1e-7, we identified 9 TFs with potential regulatory relationships in 7 target genes from Spd, 5 TFs possess over-represented targets, 14 TFs with potential regulatory relationships in nine target genes from FA, and 3 TFs possess over-represented targets (**Supplementary Table 5**).

**Figure 10:**
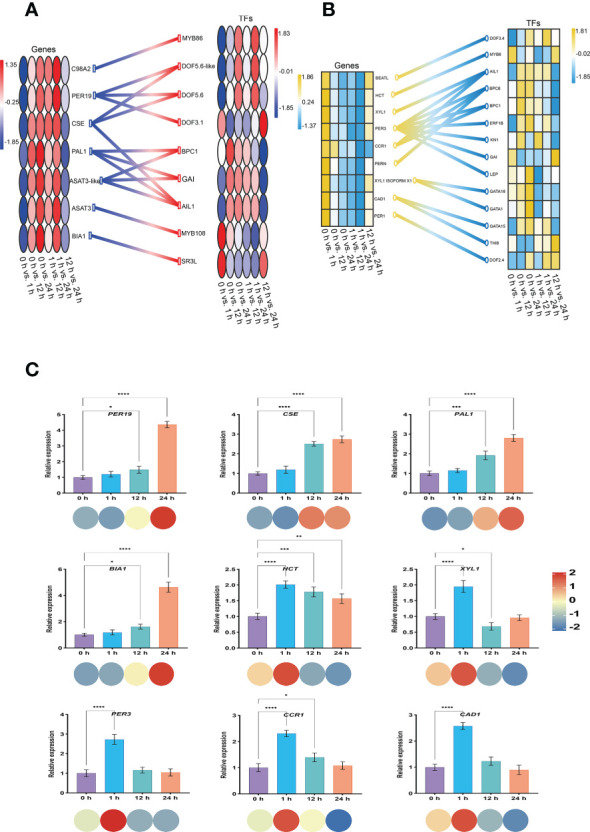
Construction of a regulatory network of genes and TFs and qRT-PCR analysis. **(A)** Heatmap of Spd synthesis genes and regulatory TFs involved in *S. lycopersicum* roots in response to salt stress. **(B)** Heatmap of FA synthesis genes and regulatory TFs involved in *S. lycopersicum* roots in response to salt stress. **(C)** qRT-PCR analysis of the genes involved in Spd and FA synthesis in *S. lycopersicum* roots in response to salt stress. The heatmap of genes is located on the left side, and the heatmap of TFs is located on the right side. Different colored circles or boxes represent the RNA-seq expression profiles of the genes, and the connecting lines between genes and TFs represent regulatory relationships. The bars represent the qRT-PCR expression of the genes, and the corresponding circles of different colors below represent the RNA-seq expression profiles of the genes. Three independent biological replicates were used to calculate the mean. Error bars indicate the SD of the three biological replicates. Values indicate mean ± SD. Dunnett’s multiple comparison test was applied to verify the significance of differences (^*^ *P* < 0.05, ^**^ *P* < 0.01, ^***^ *P* < 0.001, and ^****^ *P* < 0.0001).

Generally, the expression of genes associated with the regulation of Spd synthesis in the *S. lycopersicum* roots obtained from the screening progressively increased with the extension of salt stress treatment time, while the expression of genes associated with the regulation of FA synthesis was progressively less. We constructed schematic diagrams of the TFs engaged in the gene regulation of Spd and FA synthesis in response to salt stress in *S. lycopersicum* roots, respectively, to gain a clearer insight into the regulatory relationships between the two in the relevant pathways under salt stress (**Figures 10A** **, B**). The findings indicated that the BPs of Spd and FA are regulated by most TFs, including the common MYB, Dof, BPC, GRAS, and ERF. Meanwhile, some genes are regulated by only one TF, while some genes are regulated by 2-3 TFs. In particular, *PER3* in the regulation of genes related to FA synthesis is regulated by 7 TFs. Finally, we randomly performed qRT-PCR analysis on a total of nine genes involved in Spd (*PER19*, *CSE*, *PAL1*, and *BIA1*) and FA (*HCT*, *XYL1*, *PER3*, *CCR1*, and *CAD1*) synthesis to examine the reproducibility and accuracy of the RNA-seq outcomes (**Figure 10C**). The results showed that the expression of the genes in each sample was generally in agreement with the transcriptome outcomes, except for *HCT*, suggesting that the transcriptome outcomes were credible.

## Discussion

The global challenges of environmental stress (including soil salinization) and climate extremes are expected to cause significant changes in soil properties in the coming years, with the expansion of persistent salinization adversely affecting the global area of arable land while also impeding plant growth and development and high crop yields and quality (Butcher et al., 2016; Chele et al., 2021).

Currently, various omics techniques have become an effective strategy to better understand the response mechanisms to environmental stresses such as salt stress (Dai et al., 2022). In recent years, there have been many reports on the study of salt tolerance in plants through transcriptomic approaches (Chen et al., 2022a; Ge et al., 2022; Wang et al., 2022a; Xu et al., 2022). Recently, metabolomic studies on salt tolerance in plants have reported that increased accumulation of metabolites and related pathways that mainly regulate the antioxidant defense system in different Indian mustard (*Brassica juncea*) varieties under different periods of salt stress give the high-yielding variety CS60 an advantage over CS245-2-80-7 and may be the main tolerance mechanism for its resistance to salt stress (Singh et al., 2022). *Bacillus* sp. mediated salt tolerance studies in wheat (*Triticum aestivum*) showed that inoculation with wp-6 promotes the growth of wheat seedlings under salt stress mainly by regulating amino acid metabolism, porphyrin metabolism, and chlorophyll metabolism (Zhao et al., 2022). The expression of L-fucose and succinate, two key nodes of metabolites in the salt stress regulatory network, were up- and down-regulated, respectively, suggesting that they may be important for pecan (*Carya illinoinensis*) to adapt to salt stress (Jiao et al., 2022). Barley (*Hordeum vulgare*) seeds respond more positively to salt stress at the metabolic level during germination compared to salt-sensitive materials. Salt-tolerant germplasm may enhance stress resistance by repairing intracellular structures, promoting lipid metabolism, and increasing osmotic metabolites (Chen et al., 2022b).

At the same time, a more in-depth and systematic study of salt tolerance mechanisms in plants is possible based on the combination of omics approaches. A total of 103 key DAMs and 53 key DEGs that may be associated with salt stress were identified in two *B. napus* varieties with significant differences in salt tolerance using a combined transcriptomic and metabolomic analysis, revealing the regulatory mechanisms of salt tolerance in *B. napus* and providing rich data for further in-depth identification of essential genes for salt tolerance in *B. napus* (Wan et al., 2022). A combined transcriptomic and metabolomic analysis showed that genes involved in phenylpropanoid biosynthesis, phytohormone signaling, and carbohydrate biosynthesis pathways, especially bHLH family TFs, played an important role in improving salt tolerance in watermelon (*Citrullus lanatus*) seedlings after exogenous alglucan treatment (Yuan et al., 2022). The combined transcriptomic and metabolomic findings suggest that cell wall remodeling and adenosine triphosphate-binding cassette transporters are involved in the response of strawberry (*Fragaria* × *ananassa*) to salt stress, which provides new insights into the potential molecular mechanisms of *F.* × *ananassa* response to salt stress and offers potential targets for breeding salt-tolerant *F.* × *ananassa* varieties (Li et al., 2022).

*S. lycopersicum* is a moderately salt-sensitive plant and seedlings are vulnerable to severe impacts of salt stress during the growth period (Cuartero et al., 2006; Tanveer et al., 2019). Roots, as the main organs of plants to perceive salt stress signals, play an essential function in response to salt stress (Deolu-Ajayi et al., 2019; Nakamura et al., 2020). Therefore, an integrated metabolomic and transcriptomic analysis to investigate the response of *S. lycopersicum* seedling roots to salt stress may be meaningful to reveal the mechanism of salt tolerance in *S. lycopersicum*.

### Phenotypic and physiological responses of *S. lycopersicum* during different salt stress periods

In the present study, we first conducted an experimental study on the phenotypic and physiological index changes of *S. lycopersicum* under salt stress to observe and study the effects of salt stress on *S. lycopersicum*. Phenotypic analysis of *S. lycopersicum* leaves under different treatment periods of salt stress showed that *S. lycopersicum* developed some adaptations after 12 h of salt stress, and the leaf and petiole phenotypes were partially restored to normal, probably through self-regulation to reduce the damage induced by salt stress.

MDA, a substance of lipid peroxidation, is a symbol of plasma membrane disruption (Pan et al., 2017). Although the MDA content in *S. lycopersicum* roots under salt stress was always highly significantly greater than the control, the increase from 12 h to 24 h was smaller, suggesting that *S. lycopersicum* slowed down the damage to its plasma membrane caused by salt stress during this period. The accumulation of Pro not only acts as an osmoregulatory factor in the plant cytoplasm, but also serves as an essential function in stabilizing the structure of biomolecules, reducing cell acidity, and regulating cell redox (De la Torre-Gonzalez et al., 2018). The Pro content in *S. lycopersicum* roots under salt stress was significantly higher than that of the control at 12 h and 24 h, with a greater increase from 1h to 12 h. This may indicate that *S. lycopersicum* is subjected to a gradual increase in osmotic protection under salt stress, while it is relatively weak at the initial of the stress.

At the same time, the results of the physiological indexes examined in the roots under different treatment periods of salt stress showed that salt stress induced a continuous and large production and accumulation of intracellular H_2_O_2_ and O_2_^-^, which are important representatives of ROS (Kashyap et al., 2020a). However, the content of H_2_O_2_ increased the least from 1 h to 12 h, while the content of O_2_^-^ had shown a decreasing trend after 12 h of salt stress, which was related to the effective activation of the mechanism of ROS scavenging in plants.

Typically, salt stress can increase oxygen-induced cellular damage due to increased ROS production. Therefore, salt tolerance may depend, at least in part, on the enhancement of the antioxidant defense system, which includes antioxidant compounds and several antioxidant enzymes (Waseem et al., 2019). In the present study, the response of SOD, POD, CAT, APX, and GR activities indicated that oxidative stress is an important component of salt stress in tomato plants.

SOD is an important constituent member of the antioxidant enzyme family in biological systems and plays a key role in the cellular defense mechanism against ROS. Its activity regulates the relative levels of H_2_O_2_ and O_2_^-^, reducing the risk of free radical formation and the consequent possible damage to membranes, proteins and DNA. The active product of SOD is H_2_O_2_, which is still toxic and must be eliminated by conversion to water in subsequent reactions. In plants, many enzymes regulate intracellular levels of H_2_O_2_, such as POD, CAT and APX, three important classes of enzymes (Alscher et al., 2002; Wang et al., 2017).

POD uses H_2_O_2_ as an electron acceptor to directly oxidize phenolic or amine compounds, with the dual effect of eliminating H_2_O_2_ and phenolic amine toxicity, and can work synergistically with SOD and CAT to scavenge excess free radicals in plants, which is one of the key enzymatic defense system in plants under adverse conditions (Passardi et al., 2005; Wu et al., 2019). The main role of CAT is to catalyze the decomposition of H_2_O_2_ into water and oxygen and to remove H_2_O_2_ from plants, thus saving cells from H_2_O_2_ poisoning, and is one of the key enzymes of biological defense system (Anjum et al., 2016). APX is an important antioxidant enzyme for ROS scavenging in plants and a key enzyme of the AsA-GSH cycle. It catalyzes the H_2_O_2_ dependent oxidation of L-ascorbic acid, and by regulating the production of APX can modulate redox signaling in cells and thus improve plant tolerance to abiotic stresses (Pandey et al., 2017).

Our results showed that POD and CAT activities changed in line with SOD activities with the extension of salt stress treatment period, both of which increased significantly and showed an overall increasing trend followed by a decreasing trend, reaching a peak at 12 h after stress. This suggests that the ROS scavenging mechanism was effectively activated at the initial stage, but could not continue to maintain the redox homeostasis at the later stage. After salt stress, APX activity did not change significantly at 1 h and 12 h but decreased significantly at 24 h. It is possible that the high concentration of H_2_O_2_ at the later stage destroyed the intermediate products of APX catalytic process, which led to the inhibition of APX activity. SOD is largely coordinated with CAT, POD, and APX activities at the beginning of salt stress, while this dynamic equilibrium is gradually disrupted at later stages. They play a central protective role in the scavenging of H_2_O_2_ and O_2_^-^, and the active participation of these enzymes is at least partially related to the oxidative stress tolerance of tomato plants at the beginning of salt stress.

GR, as an antioxidant enzyme, protects plants from oxidative stress by participating in the plant AsA-GSH cycle and interacting with SOD, APX, and glucose-6-phosphate dehydrogenase to scavenge excess ROS in plants, thus allowing them to survive in adversity (Rahantaniaina et al., 2017; Muller-Schussele et al., 2020). Usually, the variation of GR enzyme activity varies with species, variety, and stress conditions (Madhu et al., 2022). We found that GR activity decreased slightly but not significantly after salt stress, and considering that the significant decrease in APX activity also occurred only at a later stage, it is speculated that the AsA-GSH cycle exhibits lower activity in tomato roots and that the Halliwell-Asada enzyme pathway may also play a role in the initial salt tolerance of tomato roots.

The phenotypic changes observed above are consistent with the trends of physiological indices, which provide a basis for further investigation and analysis of salt resistance mechanisms in *S. lycopersicum*.

### Adaptation strategies of the multi-omics in *S. lycopersicum* roots to salt stress

Plants adapt to salt stress *via* complex regulatory mechanisms (Zhu, 2002; Yuan et al., 2016; Zhu, 2016; Ma et al., 2022). In recent years, combined metabolome and transcriptome analysis methods have been broadly applied to investigate the effects of salt stress on rice (*Oryza sativa*) (Wang et al., 2021b), oat (*Avena sativa*) (Xu et al., 2021), sesame (*Sesamum indicum*) (Zhang et al., 2019), shrub (*Nitraria sibirica*) (Li et al., 2021), and wheel wingnut (*Cyclocarya paliurus*) (Zhang et al., 2021; Zhang et al., 2022) and their regulatory mechanisms in response to salt stress. Although there have been numerous investigations on the mechanisms of response to salt stress in *S. lycopersicum*, most of these studies have been limited to the physiological and ecological levels or a single omics pathway (Rouphael et al., 2018; Kashyap et al., 2020b; Zhu et al., 2022). Because the mechanisms by which genes express metabolites are influenced by multiple factors, an increase or decrease in gene expression does not necessarily lead to a decrease or increase in metabolites. Thus, salt stress may have led to sizable alterations in the transcriptome, changing metabolites, and related pathways that regulate tolerance (Mu et al., 2021). In addition, little information is known about the relationship between transcriptional and metabolic responses to salt stress in *S. lycopersicum*, and some relevant studies still focus on the leaves of *S. lycopersicum* (Mellidou et al., 2021).

Since the effective response capacity of plants under salt stress depends on the roots (Deolu-Ajayi et al., 2019; Nakamura et al., 2020). A combined metabolome and transcriptome analysis have been carried out in *G. max* (Jin et al., 2021), *S. alopecuroides* (Zhu et al., 2021), *B. vulgaris* (Liu et al., 2020), and *B. napus* (Wang et al., 2022b) roots to reveal the molecular mechanisms underlying their salt tolerance. In the present study, based on the morphological and physiological responses of *S. lycopersicum* to salt stress, metabolomic and transcriptomic approaches were used to analyze the DEGs and DAMs of *S. lycopersicum* roots during various stages of salt stress and to identify significantly enriched KEGG pathways, mainly “biosynthesis of secondary metabolites”, “plant hormone signal transduction”, and “phenylpropanoid biosynthesis”. This is consistent with the results of former works that used a multi-omics approach to analyze the response mechanisms and metabolic processes of *S. alopecuroides*, *B. vulgaris*, and *B. napus* roots to salt stress (Liu et al., 2020; Zhu et al., 2021; Wang et al., 2022b), but also differs somewhat from the results of studies on the molecular mechanisms of salt tolerance in *G. max* roots (Jin et al., 2021).

During their long-term evolution, plants have gradually developed some physiological and ecological functions adapted to their environment, one of which is the production of various types of secondary metabolites according to the needs of incipient growth (Kutchan, 2001; Sohn et al., 2022). Secondary metabolites are the result of the interaction between plants and their living conditions during long-term evolution and can accumulate in plants to resist biotic and abiotic stresses, but also occupy an essential position in the whole metabolic activities of plants, and their production and distribution are usually species-, organ-, tissue-, and growth and developmental stage-specific, and many plant secondary metabolites are necessary for plant life activities. The synthesis and storage sites, transport pathways, metabolic dynamics regulated by developmental and environmental factors, metabolic molecular regulation, chemical ecological significance, and physiological effects of secondary metabolites in plants have been extensively investigated (Shitan, 2016; Kessler and Kalske, 2018; Yang et al., 2018a; Pang et al., 2021). Phytohormones are key regulators of signal transduction pathways such as auxin, cytokinins, abscisic acid (ABA), gibberellins, and ethylene. Phytohormones are involved in almost every process that regulates plant growth and development, both in terms of regulating the growth and development of the plant itself, and in regulating its adaptation to the environment by interacting with the external environment in which the plant lives, and playing an essential function when the plant is subjected to biotic and abiotic stresses. Currently, the synthesis, transport, signal transduction, and degradation of phytohormones and their mechanisms of action in plant growth are relatively well understood (Emenecker and Strader, 2020; Khan et al., 2020; Waadt, 2020; Kapoor et al., 2021).

Phenylpropanoids perform an active role in the growth and development of plants and response to adversity stress and are also closely related to people’s production life. With the discovery of a large number of biologically active phenylpropanoids, phenylpropanoid biosynthesis and regulation have become a research hotspot (Deng and Lu, 2017; Sreekumar et al., 2022). The phenylpropanoid biosynthetic pathway is one of the three major secondary metabolic pathways in plants, which starts with phenylalanine, catalyzed by PAL to produce cinnamic acid, C4H to p-hydroxycoumaric acid, and 4CL to 4-coumaric acid coenzyme A. This leads to the downstream specific synthetic pathway to different phenylpropanoid metabolites, including lignans, flavonoids, coumarins, terpenoids, anthocyanins, etc (Yao et al., 2021). Phenylpropanoids are ubiquitous in plants, and these secondary metabolites, which share a hydroxyl aromatic ring (Boudet, 2007; Garibay-Hernandez et al., 2021), play an active function in plant growth and development processes and in response to adversity stresses, such as acting as plant antitoxins, stress protection factors, signaling molecules, generating flower and fruit color, and cell composition (Baxter and Stewart, 2014; Kim et al., 2020; Bauters et al., 2021). We discovered that the pathway of phenylpropanoid biosynthesis in *S. lycopersicum* roots changed significantly under different salt stress periods, and the DEGs and DAMs obtained in this pathway were highly correlated, and the analysis of this regulatory pathway is important for studying the molecular mechanism of *S. lycopersicum* response against salt stress.

### FA and Spd in the phenylpropanoid biosynthesis pathway are associated with salt stress

Our pathway analysis, correlation analysis, and CCA results showed that FA and Spd in the phenylpropanoid biosynthesis pathway were highly associated with the corresponding DEGs, respectively. FA is a phenolic hydroxyl-containing phenylpropanoid natural product, which is a kind of phenolic acid, produced by the biosynthesis of lignin from phenylalanine or tyrosine. It is a “bridge” for covalent cross-linking between hemicellulose molecules and between hemicellulose and lignin molecules and performs a key function in the formation of the complex heterogeneous polymer network structure of plant cell wall and its lignification process. It makes the cell wall strong and impedes the degradation of the cell wall by lignocellulose-degrading enzymes such as cellulase and xylanase, and is an important molecular basis for the formation of cell walls antidegradation barrier (Yu et al., 2005; Antonopoulou et al., 2021). Meanwhile, FA has strong antioxidant properties and can scavenge H_2_O_2_, hydroxyl radicals, O_2_^-^, and peroxynitrite very well. It also has a physiological modulating effect, not only inhibiting free radical-producing enzymes but also increasing the activity of free radical-eliminating enzymes (Mathew and Abraham, 2004; Berton et al., 2020). Lignin is the second most abundant secondary metabolite in nature following cellulose and important structural material in plant cell wall formation, which has important biological functions for the plant body and can improve plant resistance against biotic and abiotic stresses (Chen et al., 2021). Enhancing salt tolerance to resist damage incurred by salt stress by increasing lignin content has been demonstrated in transgenic *Arabidopsis thaliana* (Duan et al., 2020), apple (*Malus × domestica*) (Chen et al., 2019; Chen et al., 2020), sweet potato (*Ipomoea batatas*) calli (Park et al., 2011), and Chinese cabbage (*Brassica rapa*) (Cao et al., 2020). In the present study, we discovered that some genes engaged in FA synthesis were significantly up-regulated in the 0 h vs. 1 h comparison group at the beginning of salt stress, and the respective metabolites were as well up-regulated. This result implies the possibility that *S. lycopersicum* roots could improve their resistance to salt stress *via* enhancing FA content and offers a basis for further identification of salt tolerance genes.

At the same time, plants have developed a variety of strategies to deal with stress over a long time, one of which is to protect cellular osmotic pressure through soluble substances, sustain intracellular water balance, and keep the stability of cell membranes (Karnik et al., 2017). Spd is produced by transferring one molecule of aminopropyl to putrescine by Spd synthase, which is a group of low molecular mass aliphatic nitrogenous bases that perform critical functions in plant growth, morphogenesis, prevention of senescence, and resistance to environmental stresses. It has been shown that Spd is very closely related to stress resistance in adversity, and its role in plant stress resistance is not only as a stress-protective substance but also serves as a signaling molecule in stress signal transduction, which facilitates the establishment of plant stress resistance mechanisms (Kasukabe et al., 2004; Kasukabe et al., 2006; Yang et al., 2022). Overexpression of critical enzyme genes in the Spd metabolic pathway in plants can protect against damage induced by salt stress by enhancing Spd content and enhancing salt tolerance has been demonstrated in transgenic *A. thaliana* (Kasukabe et al., 2004; Tang et al., 2021), *I. batatas* (Kasukabe et al., 2006), *O. sativa* (Roy and Wu, 2002), and tobacco (*Nicotiana tabacum*) (Waie and Rajam, 2003). Meanwhile, some reports on the exogenous application of Spd to enhance salt resistance in mung bean (*Vigna radiata*) (Nahar et al., 2016), *O. sativa* (Roychoudhury et al., 2011), ginseng (*Panax ginseng*) (Parvin et al., 2014), and *S. lycopersicum* (Raziq et al., 2022) have also been reported. In the present study, we observed that Spd content was substantially up-regulated in all comparison groups except 0 h vs. 1 h and 12 h vs. 24 h, presumably making an important contribution during the mid- to late-stress response to salt stress in *S. lycopersicum*. This suggests that *S. lycopersicum* roots may improve their salt tolerance by increasing Spd content, which offers a basis for further investigation of the salt resistance mechanism. In conclusion, FA and Spd attenuated the damage induced by salt stress in *S. lycopersicum* at the early and mid-late stages, respectively, and they may be the key regulators of its salt tolerance.

### Construction of regulatory networks for gene expression and mining of TFs with potential regulatory relationships in Spd and FA

Usually, plant responses to salt stress are accompanied by alterations in the expression patterns of numerous genes, while stress intensity, duration, and other factors control the course of changes (Zhao et al., 2021; Ma et al., 2022). Our findings show that the expression of genes associated with phenylpropanoid biosynthesis was altered under different salt stress periods, producing transcriptional reprogramming effects that affected the phenylpropanoid biosynthesis in *S. lycopersicum* roots under salt stress. Phenylpropanoid biosynthesis is controlled by two main classes of genes: one is structural genes, which directly encode enzymes related to phenylpropanoid biosynthesis. The other category is regulatory genes, a class of genes that control the expression of structural genes (Deng and Lu, 2017). In general, the expression of genes associated with the regulation of Spd synthesis in the *S. lycopersicum* roots obtained from the screening gradually increased with the extension of salt stress treatment time, showing an overall increasing trend, while the expression of genes associated with the regulation of FA synthesis was suppressed, showing an overall decreasing trend.

Notably, TFs are a class of protein molecules that synergistically regulate multiple genes in the biosynthetic pathway at the transcriptional level. The phenylpropanoid biosynthesis pathway is very complex and involves a wide variety of enzymes, sometimes not controlled by one or two key enzymes, and TFs perform macroscopic functions in regulating the flow of phenylpropanoid biosynthesis through the synergistic regulation of multiple genes in the biosynthetic pathway (Meng et al., 2021).

MYB TFs are broadly present in eukaryotes and are a family of TFs with a large number and diverse functions. In recent years, with the improvement of scientific research technology, the MYB TF family has been studied more intensively in regulating the response of plants to environmental stresses (Wang et al., 2021a). The expression of many *MYB* genes responds to salt stress, for instance, overexpression of *AtMYB20* increased salt stress resistance in transgenic *A. thaliana*, while the repression lines were more susceptible to NaCl than the wild type (WT) (Cui et al., 2013). Overexpression of MYB TF of *AmRosea1* markedly enhanced the resistance of transgenic *O. sativa* to drought and salt stress (Dou et al., 2016). Numerous *MYB* genes are induced or repressed in response to salt stress and are related to the salt response of plants (Wang et al., 2021c). The Dof proteins are plant-specific TFs that are involved in various BPs during plant growth and development, such as C and N metabolism, flower and pollen development, seed development and germination, secondary metabolism, regulation of guard cell-specific genes, and vascular development (Yanagisawa, 2002; Ruta et al., 2020). SlDof22 is involved in *S. lycopersicum* ascorbic acid accumulation and salt stress response. *SlSOS1* is significantly down-regulated in RNAi lines of *SlDof22*, leading to reduced resistance to salt stress (Cai et al., 2016). Salt and low-temperature treatments induced *GhDof1* transcript accumulation. Compared with the WT, overexpression of *GhDof1* significantly enhanced the tolerance of cotton (*Gossypium hirsutum*) to salt and cold stresses (Su et al., 2017). The *BPC* gene family is a collection of plant-specific TFs that performs a critical role in the regulation of gene transcription. The *BPC* family genes are engaged in the regulation of a variety of physiological procedures in plants (Monfared et al., 2011), and although BPC is a plant-specific TF family that has been studied for a relatively short time, current studies have revealed that it can function as a bidirectional regulator of gene expression (Kooiker et al., 2005; Berger and Dubreucq, 2012). Overexpression of *CsBPC2* in *N. tabacum* harms seed germination under hyperosmotic conditions. In eukaryotes, multiple TFs usually act together to regulate gene expression (Li et al., 2019). Given that the *BPC* gene family regulates several developmental processes, it may depend on the co-action of other TFs to regulate the specific expression of target genes (Simonini et al., 2012). The GRAS TFs are plant-specific TFs engaged in plant growth and development, signal transduction, detoxification, and biotic and abiotic stress-related response processes (Waseem et al., 2022). Overexpression of *HhGRAS14* significantly increased salt and drought tolerance and reduced sensitivity to ABA in transgenic *A. thaliana* (Ni et al., 2022). Overexpression of *GmGRAS37* increased the resistance of *G. max* to salt and drought stress (Wang et al., 2020). Heterologous overexpression of *HcSCL13* increased plant growth and salt resistance in transgenic *A. thaliana* (Zhang et al., 2020). The AP2/ERF TF superfamily is one of the biggest TF families in plants and is not only participated in regulating plant growth and development and secondary metabolism but also performs a critical function in responding to stresses of adversity (Feng et al., 2020). After overexpression of *SpERF.B7*, the transgenic *A. thaliana* showed strong salt tolerance (Yang et al., 2018b). Transgenic *S. lycopersicum* and *A. thaliana* with *SlERF.B1* showed significantly higher sensitivity to salt treatment at the phenotypic and physiological levels compared with the WT (Wang et al., 2022c). Silencing of *GhERF4L* and *GhERF54L* remarkably decreased the salt tolerance of seedlings, indicating that they perform a function in regulating the response of *G. hirsutum* to salt stress (Long et al., 2019).

In the present study, we constructed schematic diagrams of TFs involved in the gene regulation of FA and Spd synthesis in response to salt stress in *S. lycopersicum* roots, respectively, to illustrate the regulatory relationships between TFs and target genes, and the findings suggested that specific TFs under salt stress can regulate the expression of phenylpropanoid biosynthesis genes *via* combining with *cis*-acting elements in the promoters of target genes. In the regulatory network, the BPs of Spd and FA are regulated by most TFs, including the shared MYB, Dof, BPC, GRAS, and AP2/ERF. Meanwhile, some genes are regulated by only one TF, while some genes are regulated by multiple TFs. Notably, we used transcriptional level analysis to explain the complex regulatory relationships between the target genes and the TFs associated with them, but further exploration and validation are needed at a later stage to identify the potential network mechanisms for enhancing salt tolerance.

## Conclusion

In summary, the present study analyzed the mechanisms of *S. lycopersicum* root adaptation to different salt stress periods by physiological, metabolomic, and transcriptomic approaches. The results showed that salt stress caused significant reprogramming of transcripts in *S. lycopersicum* roots, thereby activating secondary metabolic regulatory mechanisms in response to salt stress and that *S. lycopersicum* developed certain adaptations to salt stress by regulating the phenylpropanoid biosynthesis pathway that had already produced substantial metabolomic changes. The process of its response to salt stress may be that the rapid accumulation of ROS in a short period led to the intensification of membrane lipid peroxidation, changes in the content of osmoregulatory substances and the activities of various antioxidant enzymes to act as protective substances for membranes and enzymes and free radical scavengers, respectively, and that oxidative defense caused phenotypic changes while signal transduction activated the expression of genes related to the regulation of FA and Spd accumulation in the phenylpropanoid biosynthesis pathway, which was verified by qRT-PCR. Meanwhile, TFs such as MYB, Dof, BPC, GRAS, and AP2/ERF in the regulatory network of target genes further participated in the regulation, causing an increase in FA and Spd content at different salt stress periods, respectively, which enhanced the tolerance of *S. lycopersicum* to salt stress. Our findings not only deepen the knowledge of the molecular regulatory mechanisms of phenylpropanoid biosynthesis during salt stress but also offer a valuable basis for further improving salt tolerance in *S. lycopersicum*.

## Data availability statement

The datasets presented in this study can be found in online repositories. The names of the repository/repositories and accession number(s) can be found below: https://www.ncbi.nlm.nih.gov/, PRJNA871092.

## Author contributions

QY and JW designed the experiment and methodology. CJ and BG carried out most experimental work. BW and XL conducted data analysis. TY and NL finished plant material treatment. QY, JW, CJ, and BG wrote the manuscript. All authors contributed to experimental design and data analysis, commented on the manuscript, and gave final approval for publication.
