# Supplementary material for: Integrated metabolomic and transcriptomic analysis reveals the role of phenylpropanoid biosynthesis pathway in tomato roots during salt stress
Source: Front Plant Sci. 2022 Dec 8;13:1023696. doi: 10.3389/fpls.2022.1023696 (PMC9773889; doi:10.3389/fpls.2022.1023696)
Supplement: Supplementary file 1 [file DataSheet_1.docx]

Supplementary Material

# Supplementary Data

Supplementary Material should be uploaded separately on submission. Please include any supplementary data, figures and/or tables. All supplementary files are deposited to FigShare for permanent storage and receive a DOI.

Supplementary material is not typeset so please ensure that all information is clearly presented, the appropriate caption is included in the file and not in the manuscript, and that the style conforms to the rest of the article. To avoid discrepancies between the published article and the supplementary material, please do not add the title, author list, affiliations or correspondence in the supplementary files.

# Supplementary Figures and Tables

For more information on Supplementary Material and for details on the different file types accepted, please see [here](http://home.frontiersin.org/about/author-guidelines#SupplementaryMaterial). Figures, tables, and images will be published under a Creative Commons CC-BY licence and permission must be obtained for use of copyrighted material from other sources (including re-published/adapted/modified/partial figures and images from the internet). It is the responsibility of the authors to acquire the licenses, to follow any citation instructions requested by third-party rights holders, and cover any supplementary charges.

## Supplementary Figures

**
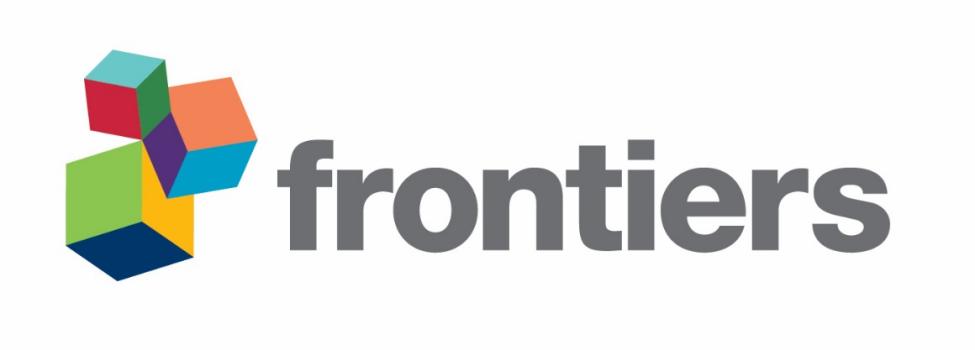
**

**Supplementary Figure 1** Volcano and bar plots of DAMs. **(A)** The comparison group of 0 h vs. 1 h. **(B)** The comparison group of 0 h vs. 12 h. **(C)** The comparison group of 0 h vs. 24 h. **(D)** The comparison group of 1 h vs. 12 h. **(E)** The comparison group of 1 h vs. 24 h. **(F)** The comparison group of 12 h vs. 24 h. Each dot in the volcano plot denotes a metabolite, where green dots denote down-regulated differential metabolites, red dots denote up-regulated differential metabolites, and gray dots denote metabolites that can be tested, but the difference is not significant. The horizontal coordinate denotes the logarithmic value (log_2_FC) of the relative content difference multiples of a metabolite in two groups of samples. The larger the absolute value of the horizontal coordinate, the larger the relative content difference of the substance between the two groups of samples. The horizontal coordinate in the bar plot is the log_2_FC of DAMs, and the vertical coordinate is the DAMs. The red bar represents the up-regulated DAMs, and green bar denotes the down-regulated DAMs.

**Supplementary Figure 2** Chord plot of DAMs **(A)** The comparison group of 0 h vs. 1 h. **(B)** The comparison group of 0 h vs. 12 h. **(C)** The comparison group of 0 h vs. 24 h. **(D)** The comparison group of 1 h vs. 12 h. **(E)** The comparison group of 1 h vs. 24 h. **(F)** The comparison group of 12 h vs. 24 h. The outermost layer in the chord plot is the name of the differential metabolite, and the scale of the middle dot represents the size of the log_2_FC value, the larger the dot, the larger its corresponding log_2_FC value. The text and the colors of the dots reflect the first level of classification of the substance, and different colors represent different metabolite source classification (class). The inner connecting lines reflect the magnitude of |r| between metabolites at corresponding positions, the red line denotes positive correlation and the blue line denotes negative correlation.

**Supplementary Figure 3** Cluster analysis graph of K means. The horizontal coordinate indicates the sample and the vertical coordinate indicates the normalized expression. The ‘Sub Class’ denotes the amount of gene classes with a similar trend, and ‘total’ denotes the total amount of genes in that class.

**Supplementary Figure 4** Z score bubble plots for different comparison groups. **(A)** The comparison group of 0 h vs. 1 h. **(B)** The comparison group of 0 h vs. 12 h. **(C)** The comparison group of 0 h vs. 24 h. **(D)** The comparison group of 1 h vs. 12 h. **(E)** The comparison group of 1 h vs. 24 h. **(F)** The comparison group of 12 h vs. 24 h. The size of the point in the bubble diagram indicates the gene count, and the vertical coordinate is *P* value, the larger the vertical coordinate, the lower the *P* value, indicating that the corresponding bubble is more remarkable. The horizontal coordinate is the up-down normalization value (the ratio of the difference between the amount of up-regulated genes and the amount of down-regulated genes to the overall differential genes). The more the value goes to the right, the greater the difference between the up-regulated genes enriched to the pathway or GO term and the down-regulated genes, and the greater the amount of up-regulated genes. The more the value goes to the left, the greater the difference in down-regulated genes and up-regulated genes enriched to that pathway or GO term, and the greater the number of down-regulated genes. On the right side is a chart of GO terms in the top 20 *P* values, with different colors representing different ontologies.

**Supplementary Figure 5** Scatter plot of KEGG enrichment analysis of DEGs. **(A)** The comparison group of 0 h vs. 1 h. **(B)** The comparison group of 0 h vs. 12 h. **(C)** The comparison group of 0 h vs. 24 h. **(D)** The comparison group of 1 h vs. 12 h. **(E)** The comparison group of 1 h vs. 24 h. **(F)** The comparison group of 12 h vs. 24 h. The vertical coordinate indicates the KEGG pathway. The horizontal coordinate denotes the rich factor (the ratio of the amount of differential genes enriched in the pathway to the amount of annotated genes). The bigger the rich factor, the higher the enrichment, and the smaller the Q-value, the more significant the enrichment. The bigger the dot, the higher the amount of DEGs enriched in the pathway. The more pronounced the color of the red dot, the more significant the enrichment. We have selected the 20 most significantly enriched pathway entries for display in this figure.

**Supplementary Figure 6** KGML reciprocal plot of combined transcriptome and metabolome analysis. **(A)** The comparison group of 0 h vs. 1 h. **(B)** The comparison group of 0 h vs. 12 h. **(C)** The comparison group of 0 h vs. 24 h. **(D)** The comparison group of 1 h vs. 12 h. **(E)** The comparison group of 1 h vs. 24 h. **(F)** The comparison group of 12 h vs. 24 h. The square pattern in the KGML reciprocal plot indicates the gene or gene product, and the rhombus pattern indicates the pathway name. Red color indicates upregulation of gene or gene product, green color indicates downregulation of gene or gene product.

**Supplementary Figure 7** Nine-quadrant plot of combined transcriptome and metabolome analysis. **(A)** The comparison group of 0 h vs. 1 h. **(B)** The comparison group of 0 h vs. 12 h. **(C)** The comparison group of 0 h vs. 24 h. **(D)** The comparison group of 1 h vs. 12 h. **(E)** The comparison group of 1 h vs. 24 h. **(F)** The comparison group of 12 h vs. 24 h. The horizontal coordinate in the nine-quadrant diagram indicates the log_2_FC of the gene, and the vertical coordinate indicates the log_2_FC of the metabolite.

**Supplementary Figure 8** Network diagram of correlations between metabolites and genes. **(A)** The comparison group of 0 h vs. 1 h. **(B)** The comparison group of 0 h vs. 12 h. **(C)** The comparison group of 0 h vs. 24 h. **(D)** The comparison group of 1 h vs. 12 h. **(E)** The comparison group of 1 h vs. 24 h. **(F)** The comparison group of 12 h vs. 24 h. The correlations between metabolites and genes were represented by network plots, and the results of the correlations between differential genes and differential metabolites in each pathway with |r| > 0.8 and *P* < 0.05 were selected for the plots. Metabolites are labeled with green squares and genes are labeled with red circles on the graph. Solid lines denote positive correlations and dashed lines denote negative correlations.

**Supplementary Figure 9** Detailed heatmap and histogram of alkaloids substances. The heatmap and histogram can show the changes of alkaloids in *S. lycopersicum* roots under different salt stress treatment times from two dimensions, thus reflecting the variability under different salt stress treatment times more visually. The heatmap is used to show the changes of the characteristic data under different salt stress treatment times, the histogram is used to show the trend percentage values of the relative content of metabolites.

**Supplementary Figure 10** Detailed heatmap and histogram of phenolic acids substances. The heatmap and histogram can show the changes of phenolic acids in *S. lycopersicum* roots under different salt stress treatment times from two dimensions, thus reflecting more visually the variability under different salt stress treatment times. The heatmap is used to show the changes of characteristic data under different salt stress treatment times, the histogram is used to show the trend percentage values of the relative content of metabolites.

## Supplementary Tables

**Supplementary Table 1** The primer sequences used in this study for qRT-PCR.

**Supplementary Table 2** The correlations between metabolites and genes in the phenylpropanoid biosynthesis pathway with |r| > 0.8 and *P* < 0.05.

**Supplementary Table 3** The CCA between DEGs and DAMs in the phenylpropanoid biosynthesis pathway.

**Supplementary Table 4** The correlations between Spd/FA and corresponding genes in the phenylpropanoid biosynthesis pathway with |r| > 0.8 and *P* < 0.05.

**Supplementary Table 5** Identification of target genes and TFs with potential regulatory relationships in Spd/FA.
