# Supplementary figures and images for: Integrated metabolomic and transcriptomic analysis reveals the role of phenylpropanoid biosynthesis pathway in tomato roots during salt stress
Source: Front Plant Sci. 2022 Dec 8;13:1023696. doi: 10.3389/fpls.2022.1023696 (PMC9773889; doi:10.3389/fpls.2022.1023696)

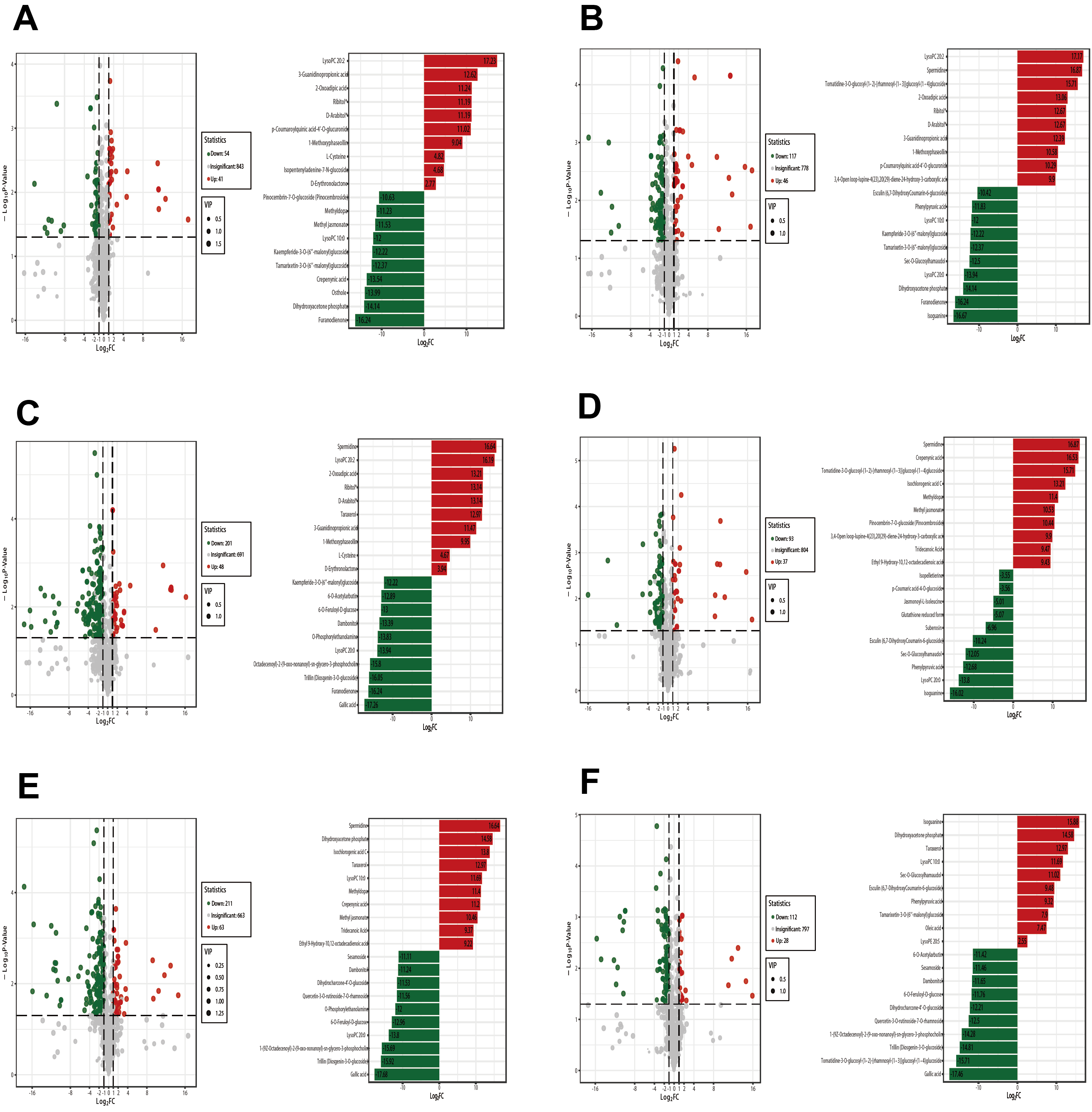

Supplement: Supplementary file 2 [file Image_1.tif]

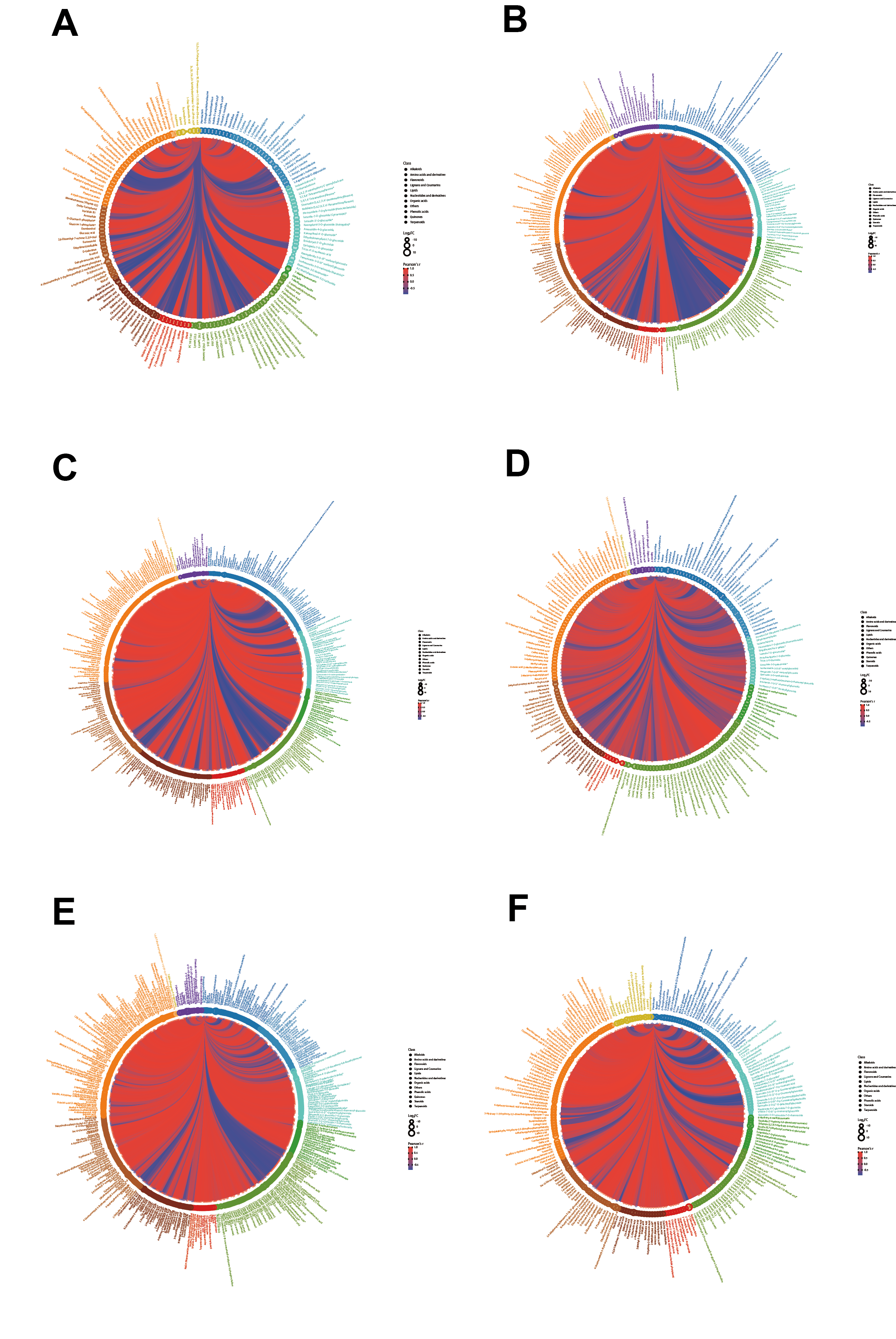

Supplement: Supplementary file 3 [file Image_2.tif]

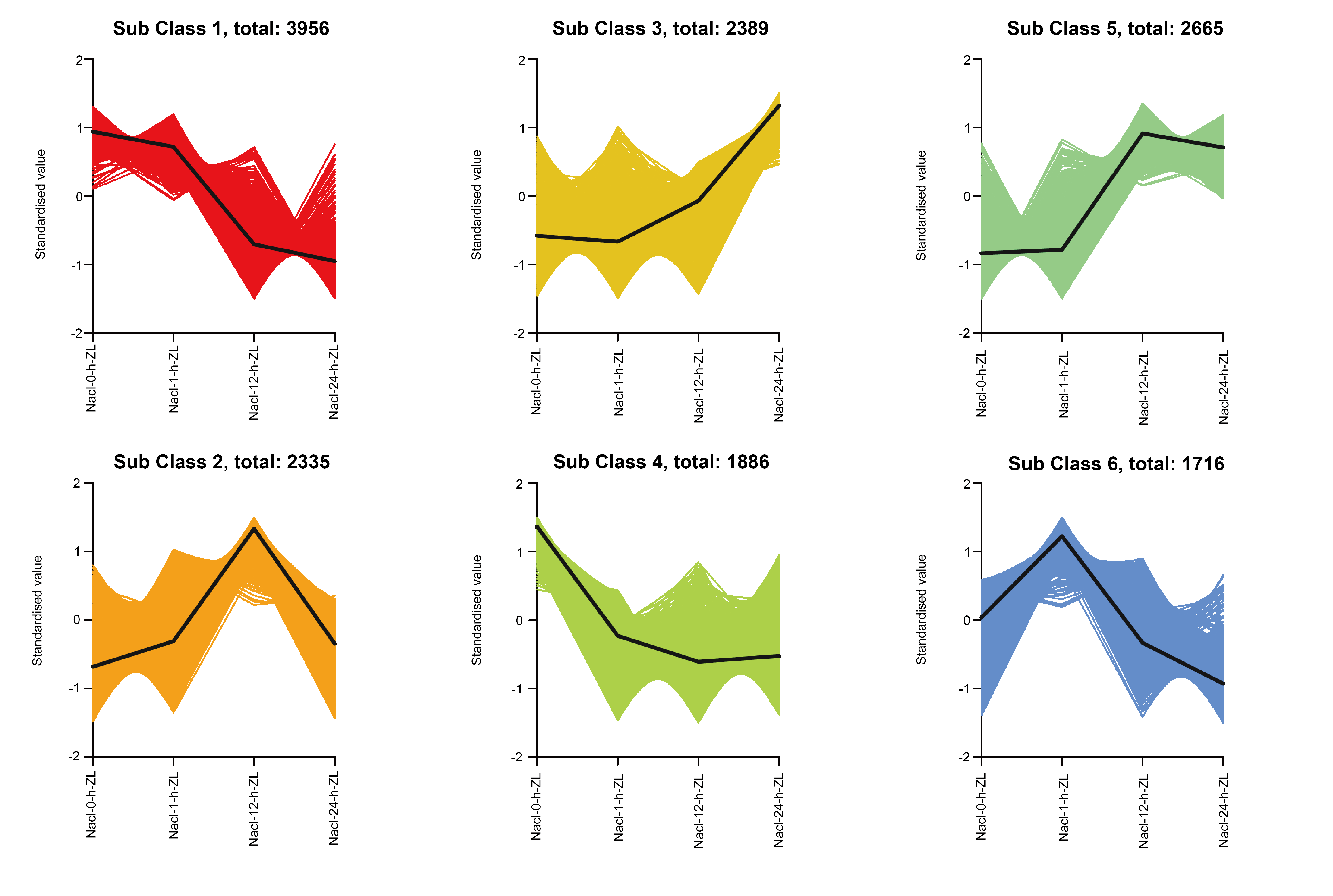

Supplement: Supplementary file 4 [file Image_3.tif]

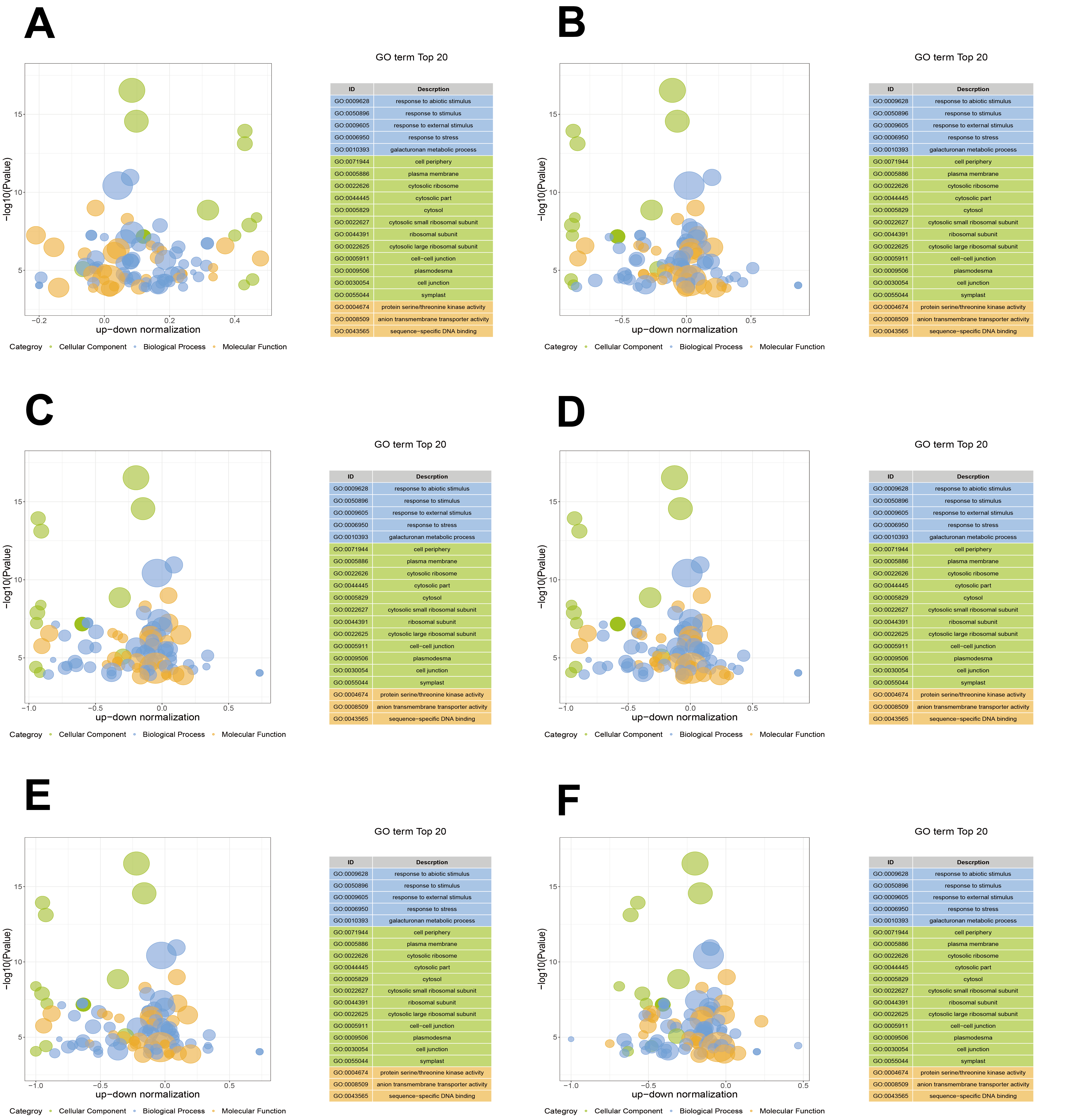

Supplement: Supplementary file 5 [file Image_4.tif]

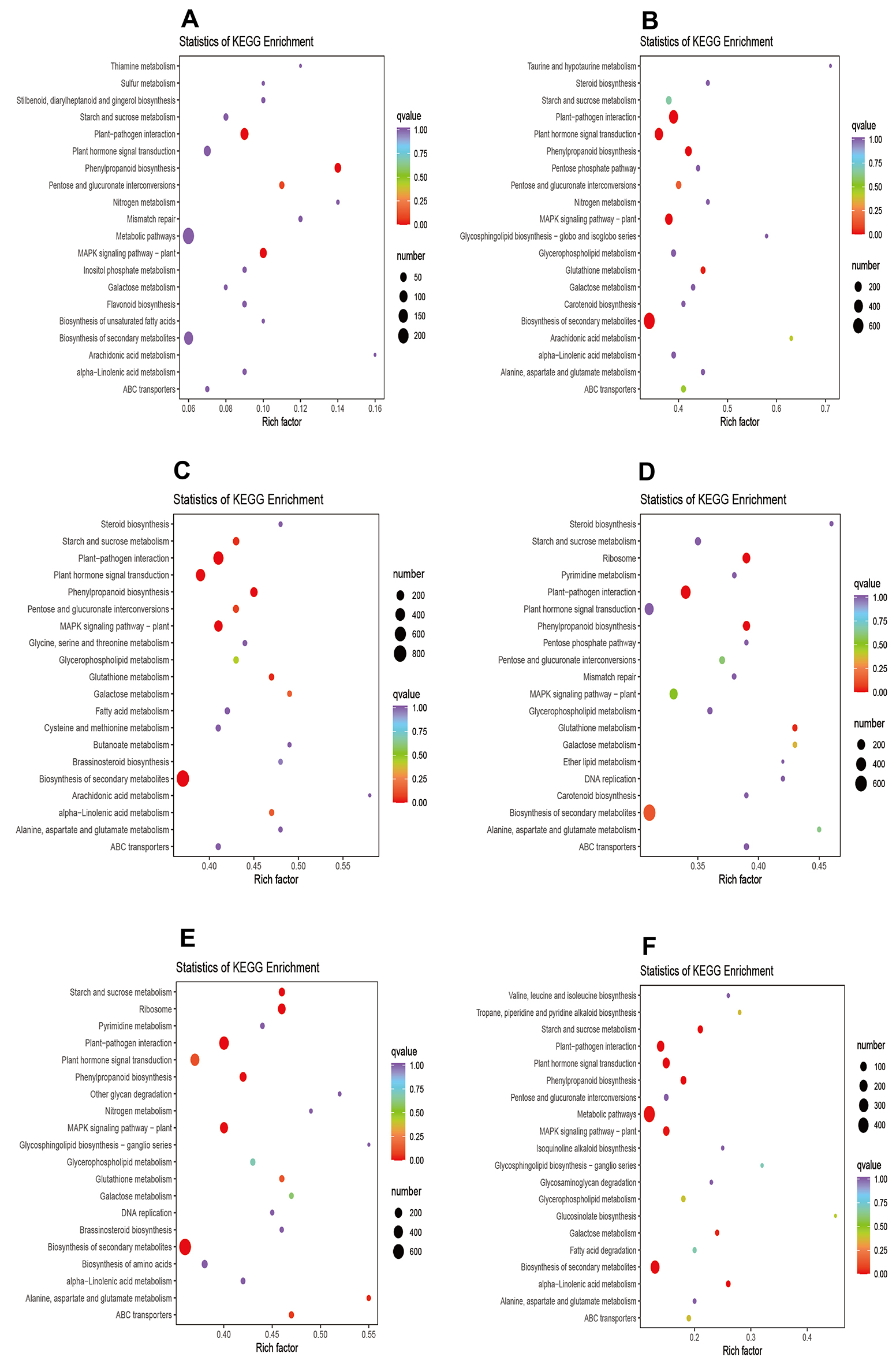

Supplement: Supplementary file 6 [file Image_5.jpeg]

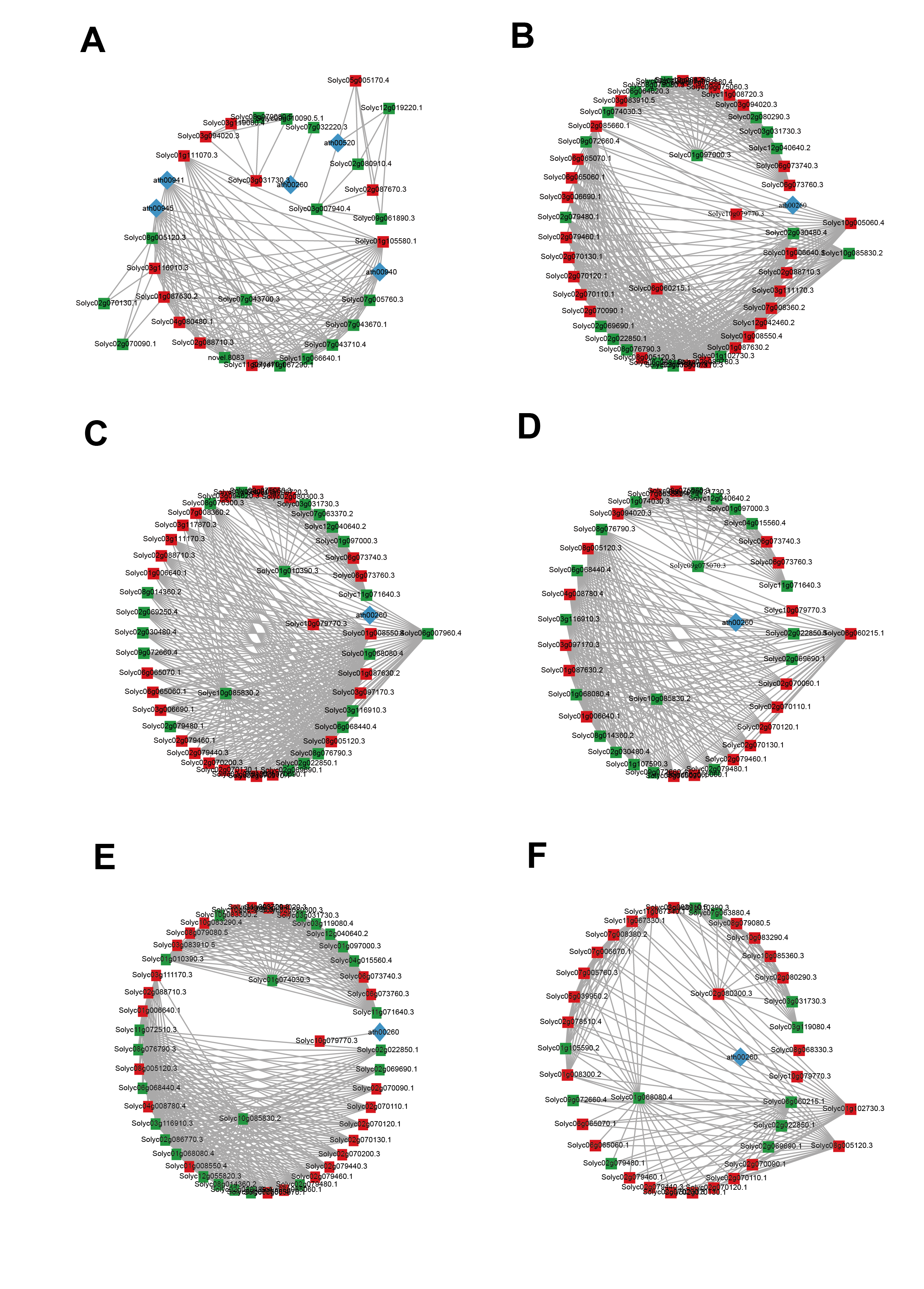

Supplement: Supplementary file 7 [file Image_6.tif]

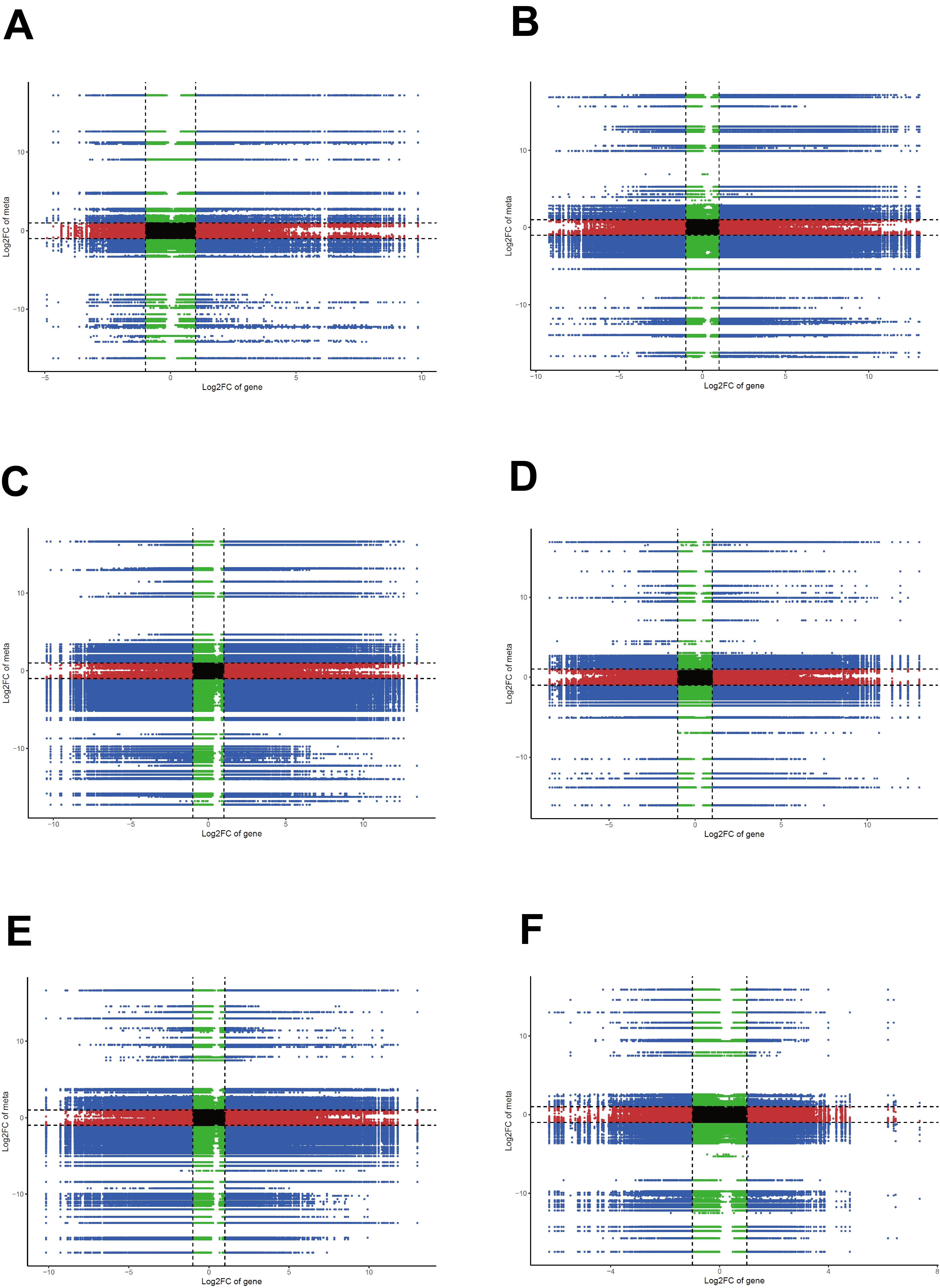

Supplement: Supplementary file 8 [file Image_7.tif]

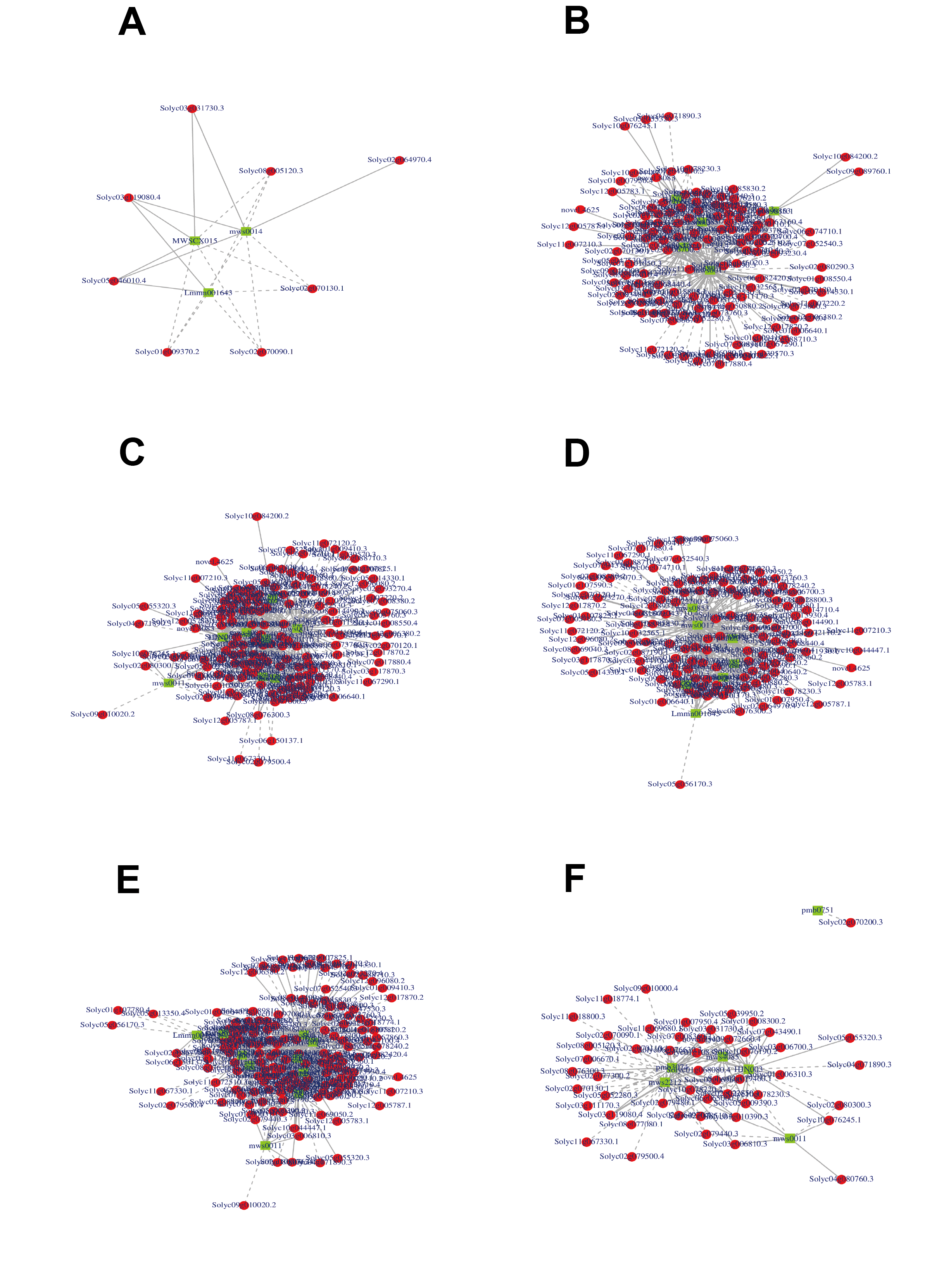

Supplement: Supplementary file 9 [file Image_8.tif]

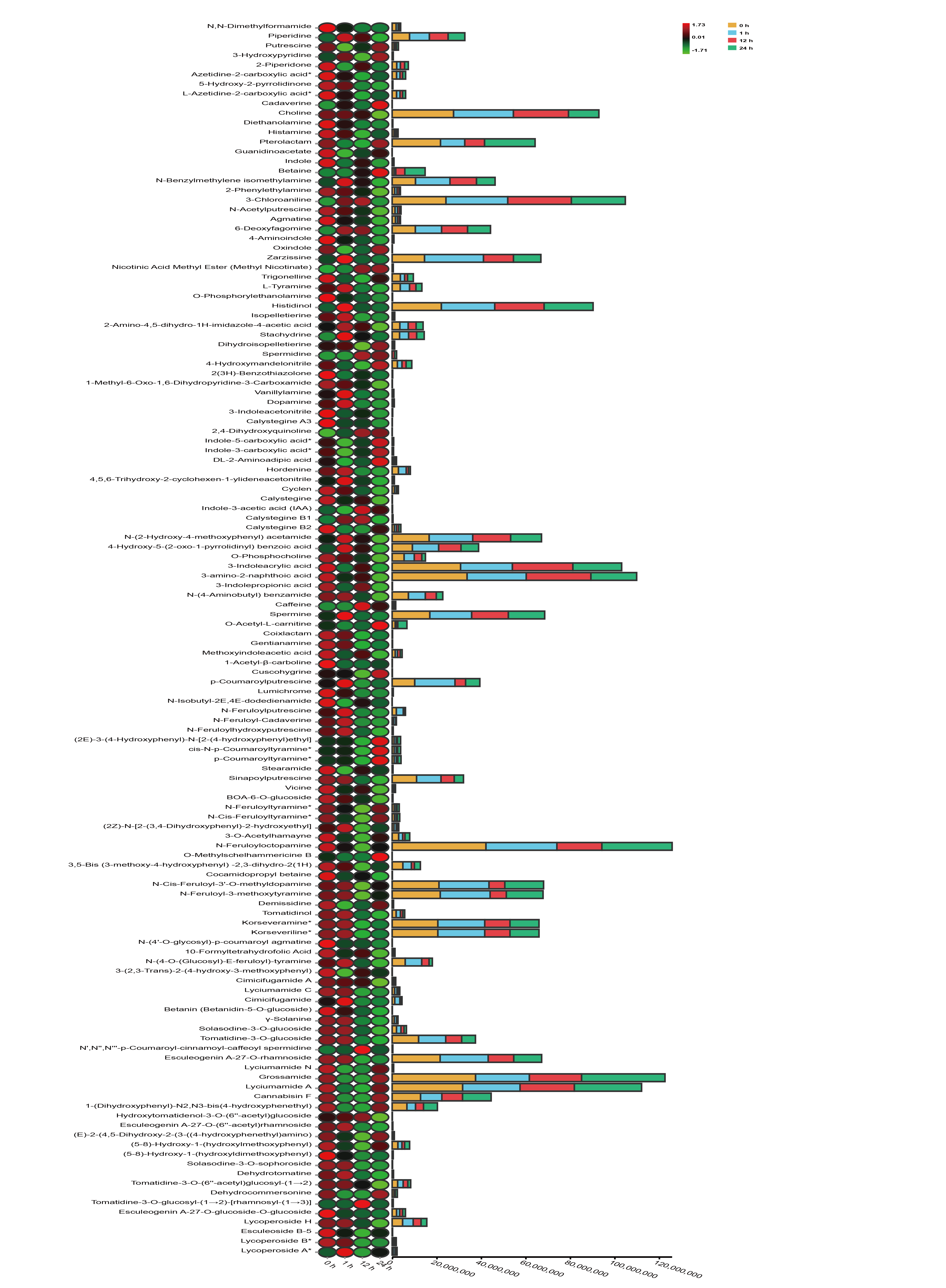

Supplement: Supplementary file 10 [file Image_9.tif]

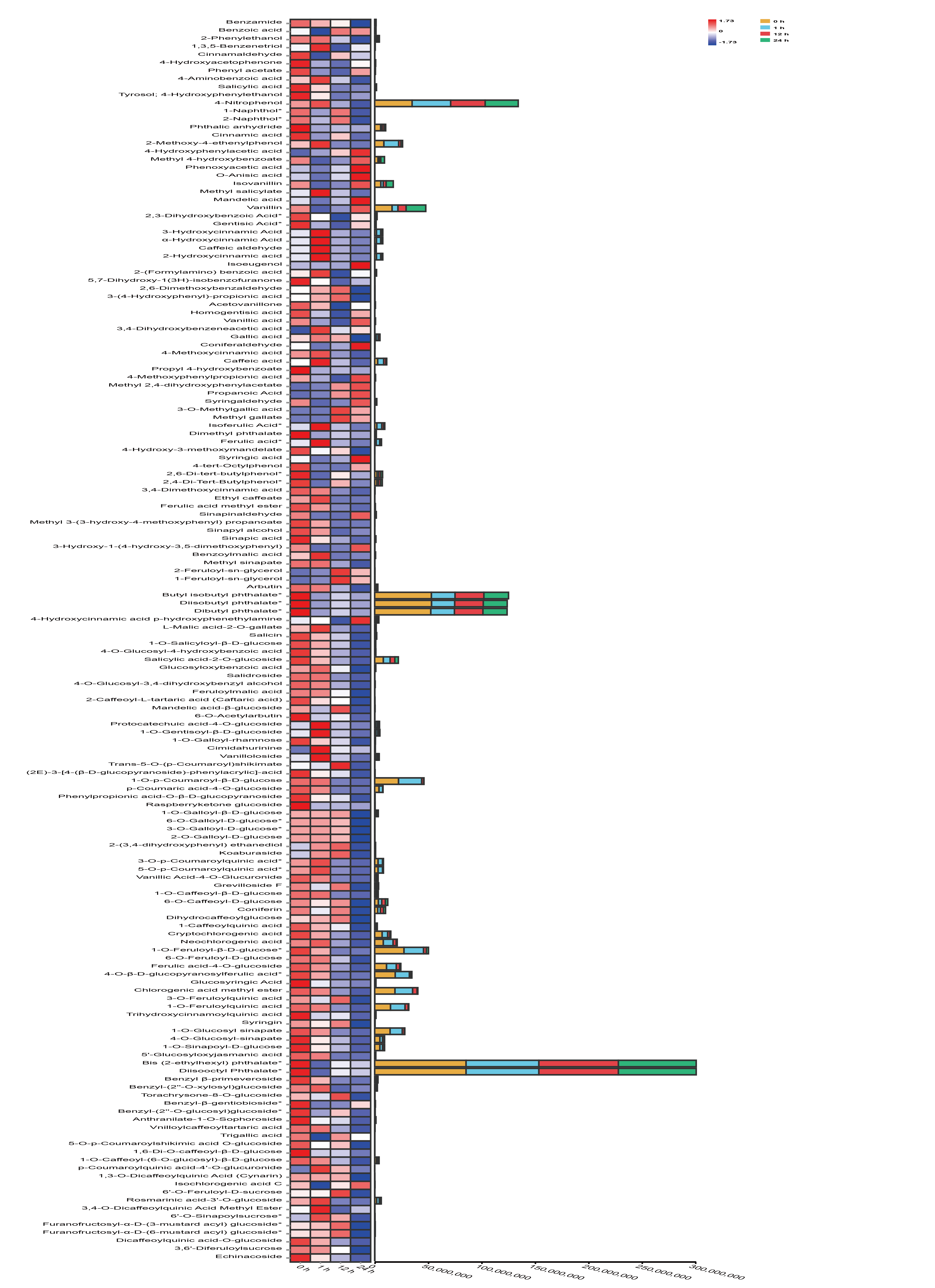

Supplement: Supplementary file 11 [file Image_10.tif]
